# Decoding brand sentiments: Leveraging customer reviews for insightful brand perception analysis using natural language processing and Tableau

**DOI:** 10.1371/journal.pone.0334330

**Published:** 2025-12-04

**Authors:** Jacqueline Jia Hsin Hu, Fahad Ahmad, Mohamed Bader-El-Den

**Affiliations:** 1 School of Computing, Faculty of Technology, University of Portsmouth, Portsmouth, United Kingdom; 2 Portsmouth Artificial Intelligence and Data Science Centre (PAIDS), University of Portsmouth, Portsmouth, United Kingdom; CCET: Chandigarh College of Engineering and Technology, INDIA

## Abstract

Traditional survey-based feedback has given way to real-time online reviews, yet transforming this unstructured text into actionable knowledge remains difficult. Focusing on the highly competitive smartphone market, where customer sentiment shapes brand perception and product strategy, this study proposes an end-to-end analytics pipeline that combines Machine Learning (ML), Deep Learning (DL), topic modelling, and interactive visualisation. Reviews for ten smartphone brands from Amazon (n ≈ 68 k) were pre-processed and class-imbalanced data were mitigated through class weighting. Sentiment classification was performed with ML models (Decision Trees, Logistic Regression, SVM, Naive Bayes) and DL models (Convolutional Neural Network (CNN), Recurrent Neural Network (RNN), Long Short-Term Memory (LSTM)). CNN achieved the highest accuracy 85.07% and balanced performance across positive and negative classes, although all models struggled with neutral reviews. Underlying themes were extracted with Latent Dirichlet Allocation (LDA) and Non-Negative Matrix Factorization (NMF); quantitative evaluation using the Coherence Score (CS) showed that NMF produced more interpretable topics (CS = 0.54) than LDA (CS = 0.41). Topic-level sentiment was assessed with the Valence Aware Dictionary and Sentiment Reasoner (VADER), linking features such as battery life and camera quality to positive or negative customer attitudes. Results are delivered through an interactive Tableau dashboard that allows practitioners to track sentiment trends, drill into coherent topics, and compare brand performance. The study also discusses ethical considerations, such as potential bias from imbalanced or culturally nuanced language, and outlines future work on cross-domain generalisation and fairness auditing. Overall, the integrated pipeline demonstrates that coupling CNN-based sentiment analysis with high-coherence NMF topics provides richer, business-ready insights than sentiment analysis alone.

## 1. Introduction

### 1.1. Background and motivation

Before the advent of the Internet, people typically visited physical stores to purchase products. Businesses could only provide feedback forms to customers for them to fill out to provide feedback on their products or customer experiences. The traditional manual feedback collection methods were often time-consuming for customers as they required filling out lengthy questionnaires and some customers might hesitate to complete the forms due to inconvenience. Hence, the data collected was limited for businesses to analyse and gain insights into customer opinions and preferences.

The rise of online e-commerce platforms has transformed this landscape. Customers can now leave reviews at any time, making feedback collection faster and more efficient. Businesses can access these reviews in real-time, allowing for quicker responses and more informed decision-making. The Internet has increased the volume and significance of customer reviews, turning them into a crucial resource. A study by Forrester found that over 30% of Internet users have shared their opinions on products online. Consumers are more likely to trust the feedback from other customer rather than relying on company advertisements [1]. Consumers gradually trust the experiences of other buyers over advertisements, which has shifted the focus of businesses toward analysing online reviews to improve customer satisfaction and product offerings.

In the rapidly growing smartphone market, brands like Samsung and Apple dominate, and e-commerce platforms like Amazon play a critical role in shaping consumer behaviour. Online reviews for smartphones have become important tools for both businesses and consumers, providing not only numerical ratings but also detailed feedback that helps buyers make informed purchasing decisions. The smartphone industry, with its constant innovation and competition, presents a unique opportunity to explore how sentiment analysis can help brands understand customer perceptions and stay competitive.

### 1.2. Problem statement

Despite the abundance of online reviews, extracting actionable insights from large datasets remains a challenge. Traditional sorting methods, such as filtering by ratings, fail to capture the nuances in customer sentiment. Sentiment analysis, powered by Natural Language Processing (NLP), addresses this by categorizing reviews into positive, negative, or neutral sentiments.

While sentiment analysis has been applied across industries, limited research exists on its application within the smartphone sector. Existing studies often lack integration with topic detection and fail to utilize interactive dashboards for deeper insights. This study bridges this gap by addressing the research question:

- How can sentiment analysis, combined with topic detection and interactive visualization, provide a comprehensive understanding of customer reviews to enhance brand perception for smartphone manufacturers?

By incorporating these techniques, this research aims to offer a holistic approach to analysing customer feedback, aiding businesses in improving brand perception and competitive positioning.

### 1.3. Aims & objective

The objective of this research is to explore the growing importance of customer reviews in the smartphone industry. By leveraging Machine Learning (ML) algorithms to analyse Amazon customer reviews, this study aims to address gaps in existing research. Amazon’s extensive consumer feedback makes it an ideal dataset for this research. Through text mining, sentiment analysis, and topic detection, this study seeks to:

- Apply sentiment analysis techniques to identify customer sentiments in online reviews.
- Implement topic detection to uncover underlying themes discussed in the reviews.
- Develop a user-friendly visualization dashboard to help businesses monitor brand perception, understand key topics, and make data-driven decisions.

This research will deepen the understanding of how customer reviews shape brand perception and provide businesses with practical tools to enhance their brand image in a competitive market.

### 1.4. Contribution and novelty

This study introduces a novel end-to-end pipeline that combines Convolutional Neural Network (CNN) sentiment classification with Non-Negative Matrix Factorization (NMF) topic detection and deploys the joint results in an interactive Tableau dashboard. Earlier work either relied on separate sentiment or topic models or required resource-intensive Large Language Models (LLMs), leaving practitioners without a single, interpretable workflow for real-time brand monitoring. By unifying CNN, NMF, and dashboard visualisation, our approach links polarity scores to specific discussion themes at scale and presents them in an operationally ready format. Beyond this technical novelty, we analyse customer reviews for ten leading smartphone brands, applying multiple ML algorithms to benchmark performance and demonstrate that our lightweight CNN-NMF pipeline achieves competitive accuracy while remaining resource efficient. The resulting interactive dashboards provide businesses with practical tools to track sentiment-topic trends, diagnose emerging issues, and tailor strategic responses. Consequently, the study advances academic understanding of integrated sentiment-topic modelling and delivers actionable guidance for industry stakeholders seeking to enhance brand reputation, customer loyalty, and product development.

### 1.5. Outline

The structure of this research is as follows: Section 1 introduces the research topic, detailing its background, objectives, and contribution. Section 2 reviews relevant literature, summarizing key findings and identifying gaps in current knowledge. Section 3 outlines the research methodology, describing the methods and techniques employed in the study. Section 4 presents the results and discussion, including a detailed analysis and interpretation of the data. Section 5 concludes with recommendations based on the research findings. The remaining sections include the references list and appendix with supplementary materials.

## 2. Literature review

### 2.1. Brand perception

Online customer reviews are closely linked to brand perception, as they directly influence how consumers view a company’s products and services. This perception develops through multiple channels such as traditional advertising, word-of-mouth recommendations, and customer service experiences. However, the influence of digital platforms has become increasingly prominent. Online reviews, social media interactions, and digital marketing campaigns now play a key role in shaping consumer perceptions to make brand management more complex.

According to [2] study, brand perception is considered a multi-dimensional construct, containing experimental, symbolic, emotional, and cognitive dimensions. This framework views brand perception through several lenses: the experimental dimension, which relates to direct interactions and experiences with the brand; the symbolic dimension, which relates to the brand’s image and associations; the emotional dimension, reflecting the feelings and emotional responses caused by the brand; and the cognitive dimension, which involves the intellectual evaluation and beliefs about the brand. Each of these dimensions contributes to how consumers form and adjust their perceptions of a brand.

Building on Keller’s framework [2], Christodoulides [3] further categorize brand perception into three critical types: brand experience, brand affect, and brand trust. Brand experience focuses on the experiential interactions consumers have with the brand, brand affect captures the emotional responses towards it, and brand trust deals with the cognitive sense of reliability and safety when engaging with the brand. These dimensions are essential for understanding consumer behaviour, especially in the context of online apparel purchases where brand perception is continually shaped by digital interactions. For instance, a brand that good in delivering positive direct experiences and consistently communicates a strong image is more likely to foster positive emotional responses and be perceived as trustworthy and dependable. These dimensions are interrelated and often reinforce one another.

In today’s digital landscape, the accessibility of online reviews allows companies to respond promptly to customer concerns and preferences, thereby strengthening customer relationships and building brand loyalty. A study by Vanani [4] found that brand communities on social networks, where customers share experiences and viewpoints, play a crucial role in shaping brand perception. Positive customer reviews can significantly enhance a brand’s reputation by amplifying favourable opinions and attract new customers. These reviews can help to build trust, reinforce brand identity, and contribute to long-term brand loyalty and success. By analysing a broad range of tweets, this study also showed that there is a significant correlation between customer support interactions and brand perception, indicating that good customer service can influence future company reputation, customer loyalty, and churn rates. Rapid and effective responses to customer inquiries and issues can lead to the improvement of customer sentiments about the brand.

### 2.2. Role of customer reviews in shaping brand perception

Customer reviews have become an essential component of brand perception as they provide insights into consumer experiences and sentiments, significantly influencing how a brand is perceived in the market. In the past, customer reviews were primarily collected through surveys or questionnaires, which provided limited data. For instance, a study [5] conducted a survey for H&M brands to analyse how social media can be used for branding purposes in Norway. The study found that client engagement with the brand was much higher on social media than through traditional methods, suggesting that brands should focus more on their social media presence. The authors also noted that the 28 questions in the questionnaire were not sufficient to cover the research subject comprehensively.

In contrast, today’s digital landscape offers more dynamic ways to collect customer reviews through social media, e-commerce platforms, and public data sources. This modern approach allows businesses to quickly adapt to consumer needs and refine their market strategies. Analysing customer opinions is crucial for companies’ global growth, as feedback serves as a valuable tool for evaluating reviews and addressing criticisms effectively [6]. Social media platforms like Twitter have become useful resources for sentiment analysis, with tools like Tweepy, a Twitter streaming API, enabling the collection of tweets on specific topics [7]. For example, research [8] used Tweepy to gather 99,850 tweets mentioning “Nike” and “Adidas” across various dates in 2018 as part of a comparative study to gauge public opinion on these leading international apparel brands. Their research revealed that most customers rely on online product reviews before making a purchase, highlighting the growing importance of public customer reviews as a vital resource for businesses aiming to improve product design and offerings. Positive reviews and high ratings significantly influence potential buyers and enhance a brand’s reputation.

Further highlighting the significance of customer sentiment reflected in reviews, study [9] conducted an aspect-based sentiment analysis on a dataset of various car brands obtained from the Kaggle website. Their findings demonstrated that a higher sentiment score is linked with an increased likelihood of consumers choosing that brand, particularly in the competitive automotive industry. These insights are helpful for brand managers and stakeholders, who can use them to sustain brand reputation and profitability by proactively addressing consumer concerns related to product reliability. They found that Lamborghini achieved the highest sentiment score among the car brands analysed, reflecting strong customer satisfaction, particularly in areas such as comfort and price justification. This illustrates how a higher sentiment score can significantly boost customer preference, with Lamborghini owners expressing satisfaction with the quality and value of their vehicles. This case highlights the critical role that positive sentiment plays in enhancing a brand’s reputation and influencing consumer choices.

### 2.3. Overview of sentiment analysis

Sentiment analysis is the process of extracting and interpreting textual data to understand its emotional content through text mining and processing techniques. NLP techniques are used in sentiment analysis to categorise sentiments as positive, neutral, or negative. This powerful tool is widely used across diverse industries such as marketing, finance, healthcare, and politics to analyse public opinion, improve product development, and support strategic decision-making. Another research [10] utilised NLP techniques to examine game reviews and categorise them into positive or negative sentiments. NLP offers several benefits in game studies, such as automated review analysis and the identification of gameplay patterns that can help improve game design. However, NLP techniques also face challenges, including difficulty in capturing language nuances such as sarcasm and irony, potential inaccuracies with context-specific language, limited generalisation of diverse player perspectives, and dependence on domain expertise for accurate interpretation.

Moreover, techniques for sentiment analysis range from lexicon-based methods to advanced ML and Deep Learning (DL) models. Lexicon-based approaches, like those used by [11] and [12] in social media sentiment analysis, assign predefined sentiment scores to words or phrases. The opinion lexicon-based method, where a lexicon consisting of positive and negative opinion words is used to evaluate sentiment in sentences as positive, negative, or neutral. This widely used method involves applying a scoring function to each sentence, based on the presence of these opinion words. These methods are simple and computationally efficient but often lack the contextual understanding needed for accurate sentiment interpretation. For example, sarcasm, irony, or idiomatic expressions can easily lead to misinterpretation because the lexicon-based approach does not account for context beyond the predefined sentiment scores. Despite these drawbacks, lexicon-based methods continue to be widely used, especially in scenarios where speed is prioritized over depth of analysis. They serve as a foundational tool in sentiment analysis, often combine with more advanced techniques to enhance accuracy.

As data grow in complexity, ML-based sentiment analysis models, such as Naïve Bayes and Support Vector Machine (SVM), offer powerful tools for analysing social media data, as demonstrated by [13–15]. These models can adapt to new data and capturing complex sentiment patterns. However, they necessitate large, labelled datasets and substantial computational resources. Their findings revealed that SVM achieved the highest predictive accuracy after cross-validation, outperforming the other models in most cases. This underscores the general observation that SVMs often deliver better performance compared to other classification models.

With the increasing volume of data, traditional classification models often face efficiency challenges, making DL models particularly advantageous for sentiment analysis. These models, such as CNN, Simple Recurrent Neural Network (RNN), and Long Short-Term Memory (LSTM), offer benefits like automatic feature extraction, faster computation with accelerated hardware, and robust performance with large datasets [16,17]. Studies show that LSTM outperforms CNN and Simple RNN due to its ability to retain word sequences and capture long-range dependencies, which are crucial for accurate sentiment analysis. While overfitting was initially a challenge, it was addressed by optimizing parameters. Another research [18] also found that LSTM outperformed lexicon-based methods for calculating brand sentiment, though its high resource demands limited analysis to shorter timeframes.

In recent years, the use of LLMs like BERT, GPT, and Llama has become increasingly common in sentiment analysis. Study [19] demonstrated the effectiveness of LLMs over traditional transformers in identifying depressive content in Bengali social media posts, yielding good outcomes. These models, which are trained on huge amounts of text, are capable of understanding and human-like language with high accuracy. Bidirectional Encoder Representations from Transformers (BERT), introduced by Google in 2018, was one of the first LLMs to make a significant impact in this area. Generative Pre-trained Transformer (GPT), known for powering tools like ChatGPT, has also gained widespread recognition. Llama2, a more recent model from Meta, builds on the foundational transformer architecture with some enhancements. A study by Kwon [20] explored the use of these models for sentiment classification, comparing their performance with traditional and DL models. The study found that BERT and GPT outperformed others, achieving an F1-score above 0.9. However, the research also highlighted challenges associated with these LLMs. For instance, training these models, required considerable computational time and resources [21]. Llama, despite being a powerful model, showed lower accuracy in this study and demanded significant resources in terms of memory and training time. Additionally, further fine-tuning Llama proved to be time-consuming, making it less practical compared to the more efficient BERT and GPT models.

Beyond sentiment analysis, topic detection plays a crucial role in interpreting large volumes of customer feedback, particularly from platforms like Twitter, where texts are often brief and incorporate slang. As short-text analysis gains prominence, [22] and [23]proposed two widely used methods: Latent Dirichlet Allocation (LDA) and NMF for clustering these texts into coherent topics. Their findings suggest that while both models are effective, NMF generally outperforms LDA, offering more distinct topic classifications in short-text analysis. Furthermore, [24] conducted a systematic review of the airline industry, applying sentiment analysis and topic modelling to demonstrate their practical utility in various industries. Integrating these techniques enables a deeper understanding of consumer feedback, proving invaluable for businesses seeking actionable insights.

### 2.4. Brand sentiment

Brand sentiment analysis focuses on evaluating consumer perceptions of a brand, informing marketing strategies, product development, and brand management. As customer feedback volume increases, sentiment classification has become a significant research area, helping businesses leverage customer opinions for better decision-making. This analysis predicts customer attitudes towards brand products and services and provides insights into consumer behaviour by systematically examining feedback to understand brand perception. Another research [13] emphasize sentiment analysis as a crucial tool for understanding customer sentiment, allowing businesses to strategically target advertisements based on positive reviews. A study [12] demonstrated the efficiency of sentiment analysis over traditional surveys by using Twitter data to create visual representations that can help businesses in understanding customer opinions. The study [25] underscore the importance of sentiment analysis in extracting actionable insights from reviews, although challenges such as sarcasm and nuanced language can affect accuracy.

The process of gathering and labelling data for ML can be time-consuming, especially with large datasets [26,27]. Correlating sentiment analysis with customer ratings helps reveal how reviews impact overall satisfaction and supports data-driven decision-making. Another research [28] used sentiment analysis at the document, sentence, and aspect levels to categorise Amazon mobile phone reviews, capturing overall sentiment, specific preferences, and detailed product features. Nevertheless, the study encountered limitations, such as the reliance on the Afinn lexicon, which may yield different results with other tools, and the manual extraction of main product features, which could vary with different methods. The research also emphasised the need for validation with other corpora and the inclusion of n-gram features and qualifiers.

In contexts like Twitter, where specific sentiment labels may be missing, Valence Aware Dictionary and Sentiment Reasoner (VADER) are an effective tool for assessing sentiment. Specifically designed for social media, VADER able to measure both the polarity and intensity of emotions, considering language tones and even emojis. Pai [29] highlighted its ability to classify tweets into positive, negative, or neutral categories based on sentiment scores, making it particularly useful for short-text analysis. Mishra [9] applied VADER in the automotive industry, finding that higher sentiment scores were linked to increased brand preference. These insights are valuable for brand managers to enhance reputation and address consumer concerns. VADER is easy to implement and does not require extensive training data, making it accessible for both researchers and businesses looking to perform sentiment analysis without needing complex ML models.

### 2.5. Synthesis and contribution

Prior investigations typically addressed sentiment analysis and topic modelling in isolation or required resource‑intensive LLMs that limit practical deployment. Few works link polarity, topic context, and decision‑support visualisation in a unified workflow. By integrating a lightweight CNN for sentiment classification with Non‑Negative Matrix Factorization (NMF) for topic extraction and presenting the joint results in an interactive Tableau dashboard, our study overcomes those fragmentation and scalability challenges. This end‑to‑end pipeline demonstrates that near‑state‑of‑the‑art accuracy, granular topic insights, and real‑time business intelligence can coexist without the computational overhead of LLMs. Consequently, the proposed method advances the state of the art by delivering an interpretable, resource‑efficient, and operationally ready framework that directly maps consumer narratives to actionable brand strategies.

## 3. Methodology

### 3.1. Data source and description

This study utilized a comprehensive dataset of Amazon reviews, obtained through Kaggle [30]for its accessibility and relevance. This dataset was selected due to its easier accessibility compared to data from social media platforms, which require payment. Additionally, Amazon is one of the world’s largest online retailers, making its review data highly comprehensive. The Amazon customer review dataset consists of two CSV files: ‘items’ and ‘reviews’. The ‘items’ CSV file provides summarised information such as Product ASIN, Product Brand, Product Name, Product URL, Product Image URL, Product Avg. Rating, Product Review Page URL, Product Total Reviews, Product Price ($), and Product Original Price ($). The ‘reviews’ CSV file contains detailed customer reviews including Product ASIN, Reviewer Name, Reviewer Rating (on a scale of 1–5), Review Date, Valid Customer Indicator, Review Title, Review Content, and Helpful Feedback. This dataset encompasses 67,987 Amazon customer reviews across ten different smartphone brands: ASUS, Apple, Google, HUAWEI, Motorola, Nokia, OnePlus, Samsung, Sony, and Xiaomi, ranging from November 24, 2003, to December 25, 2019. Moreover, the reviews in the dataset are accompanied by ratings ranging from 1 to 5, where 1 denotes the lowest rating and 5 signifies the highest. There is detailed content associated with each rating as well as information on the product and brand name. Overall, the dataset includes a total of 714 products across ten brands, making it a good source for conducting detailed sentiment analysis.

### 3.2. Data processing

Data processing involved refining the dataset by removing null values, duplicates, and unnecessary columns (e.g., URLs). The ‘items’ and ‘reviews’ files were merged using an inner join on the product “ASIN” attribute, resulting in a consolidated dataset named “Amazon_phone_review.csv”.

**Fig 1:**
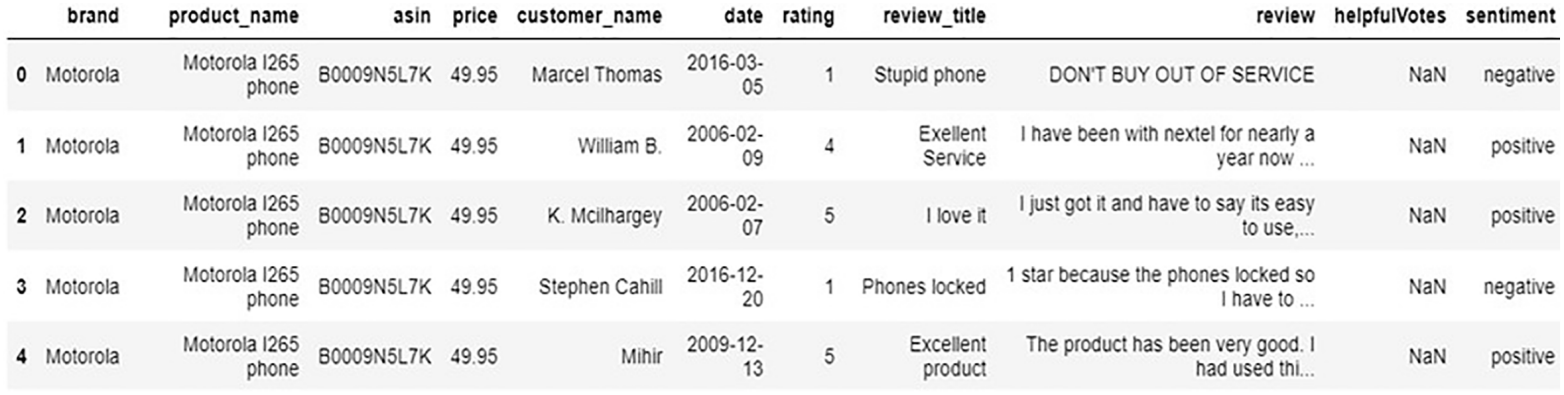
Snapshot of Merged Dataset: Amazon_phone_review.

Reviews were categorized using an automated Python-based heuristic labelling strategy: ratings of 1–2 were labelled as “Negative” 3 as “Neutral” and 4–5 as “Positive” as shown in Fig 1. This process resulted in a streamlined dataset of over 60,000 labelled reviews prepared for sentiment analysis. During model training, class weights were applied to mitigate the effects of class imbalance and ensure that underrepresented sentiment categories contributed proportionally to the loss function. This approach improved classification fairness without modifying the original data distribution. To implement this strategy, class weights were computed from the training set using the compute_class_weight function in the scikit-learn library. The resulting weight vector [Negative = 1.39, Neutral = 4.89, Positive = 0.50] reflects the inverse frequency of each sentiment class in the data. For traditional ML algorithms, these weights were supplied through the class_weight parameter (where supported) to ensure the loss function penalised mistakes on minority classes more heavily. For DL models, the same weights were passed to the fit method in Keras, thereby scaling the categorical cross-entropy loss during training.

### 3.3. Exploratory of data analysis

**Fig 2:**
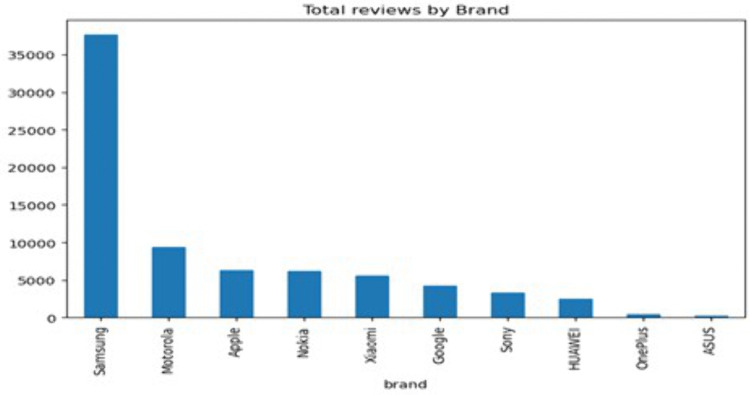
Total Reviews by Brand.

**Fig 3:**
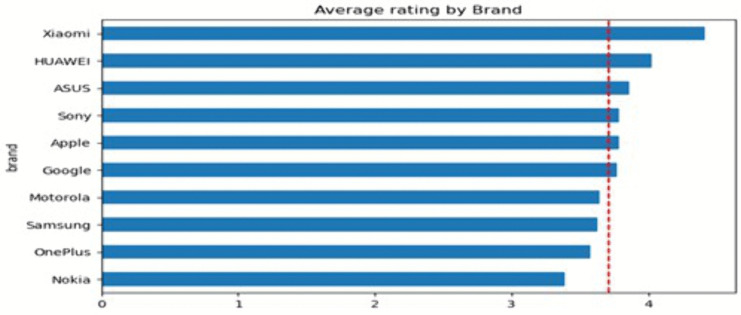
Average Ratings by Brand: The average ratings are based on a five-point scale, where 1 represents the lowest level of customer satisfaction and 5 represents the highest level of satisfaction. The red dashed line indicates the overall mean rating across all brands, serving as a benchmark for comparative evaluation.

To provide an overview of the dataset, multiple visualisations were generated using Python. First, Fig 2 reveals that the Samsung brand received the highest number of reviews, whereas the ASUS brand received the fewest. Apple, Nokia, and Xiaomi received a similar count of reviews. According to this distribution, there is a significant difference in customer engagement among brands, with Samsung dominating in terms of customer feedback volume. Next, Fig 3 shows that the average rating for all brands exceeds 3.5, with Nokia having the lowest average rating at around 3.3. While most brands have generally received positive reviews from their customers, Nokia’s products are likely to receive more critical reviews than other brands.

**Fig 4:**
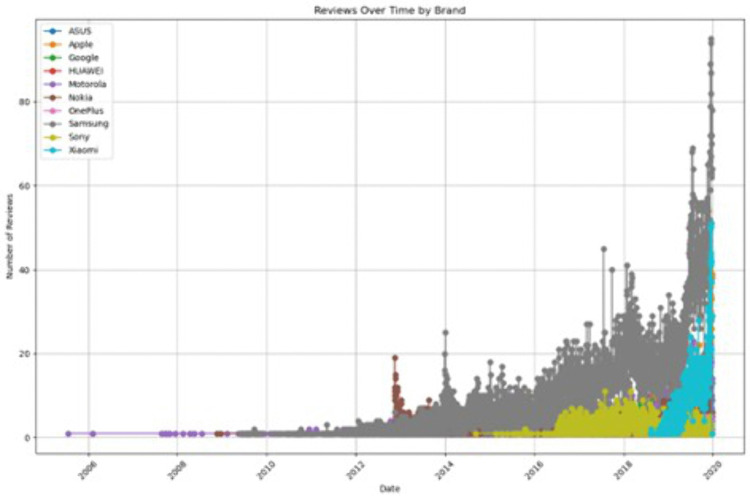
Reviews Over Time by Brand, Ranging From 2003 To 2020.

Fig 4 presents the distribution of reviews over time by brand from 2003 to 2020. Initially, from 2003 to 2008, reviews were exclusively for Motorola mobile phones. This could be due to Motorola’s early entry into the market or its popularity during that period. Subsequently, reviews began to appear for other brands, and the number of reviews steadily increased over the years. This reflects the growing diversity and competition in the smartphone market. Notably, Samsung received the most reviews overall, indicating its popularity over the years. On the other hand, in the case of the Xiaomi brand, reviews only began to appear after 2018. This late entry into the review dataset indicates that Xiaomi’s market presence significantly increased around this period. The number of reviews has continually increased since then, peaking in 2020. This trend highlights Xiaomi’s rapid growth and rising consumer interest in recent years. The detailed analysis of these visualisations provides valuable insights into customer behaviour and market trends over time, which can be crucial for strategic decision-making.

**Fig 5:**
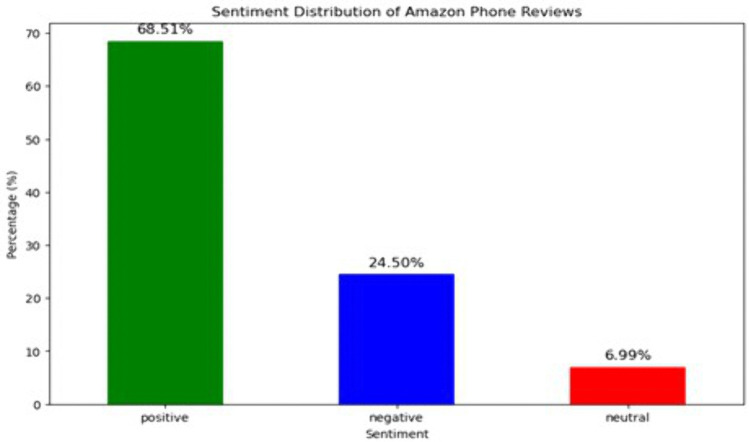
Sentiment Distribution of Amazon Phone Reviews.

Fig 5 displays the sentiment distribution of Amazon smartphone reviews. The dataset comprises 68.51 percent positive, 24.50 percent negative, and 6.99 percent neutral reviews based on the labelled ratings. To enhance statistical robustness, 95 percent confidence intervals were computed for each sentiment category using the normal approximation method for binomial proportions, from a total of over 60,000 reviews. The resulting estimates are presented in Table 1.

**Table 1:** Sentiment Distribution with 95 Percent Confidence Intervals.

Sentiment Category	Point Estimate (%)	95 Percent Confidence Interval (%)
**Positive**	68.51	68.16–68.86
**Negative**	24.50	24.18–24.82
**Neutral**	6.99	6.80–7.18

To explore brand-specific sentiment differences, data from the most recent complete year, 2019, were analysed, as all ten brands were represented with sufficient review counts. As summarised in Table 2, Xiaomi, HUAWEI, and OnePlus received the highest proportions of positive reviews above 80%, reflecting strong consumer satisfaction. In contrast, Nokia showed the highest proportion of negative reviews 36.69%, indicating comparatively lower customer satisfaction. Samsung maintained a strong positive sentiment 71.50% consistent with its market leadership, whereas Apple exhibited a higher share of negative reviews 26.95%, possibly reflecting heightened user expectations. These differences demonstrate how brand reputation and product performance are reflected in sentiment patterns within the same market year.

**Table 2:** Sentiment Distribution by Brand (Year 2019).

Brand	Positive (%)	Negative (%)	Neutral (%)	Total Reviews
Samsung	71.50	22.33	6.17	12,291
Xiaomi	84.60	10.42	4.98	4,261
Apple	66.62	26.95	6.43	3,888
Motorola	68.69	24.07	7.24	3,344
Google	65.29	27.38	7.33	2,250
Nokia	53.72	36.69	9.59	1,210
HUAWEI	82.15	12.48	5.37	1,154
Sony	66.75	24.26	8.99	779
OnePlus	81.52	14.85	3.64	330
ASUS	66.25	23.75	10.00	80

However, other sources of imbalance such as the uneven temporal and brand distribution of reviews were not explicitly addressed. For example, some brands only appear in later years (e.g., Xiaomi), which may influence the model’s ability to generalize across brands and time periods. Future work could explore temporal resampling or brand-stratified evaluation to better account for these biases.

### 3.4. Text preprocessing

Raw text data usually contains noise or irrelevant information that can impact the accuracy and effectiveness of sentiment analysis. An effective text data preprocessing is crucial for preparing raw text data for sentiment analysis. This process involves several key steps, including cleaning the data, lemmatizing with POS (part-of-speech) tagging, removing stop words, and tokenizing text. Each of these steps plays a vital role in transforming the raw data into a format that can be effectively analysed by ML algorithms.

Firstly, text cleaning is performed by removing punctuation, special characters, and numbers. This step is important because cleaning the text helps reduce noise, making the core content more accessible and analysable. Removing irrelevant information allows the model to focus on the significant features of the text, leading to better performance in sentiment analysis. Additionally, the text is standardised by converting all characters to lowercase. For instance, “BUY” or “Buy” is converted to “buy.” Without this step, words that are the same could be treated differently by text processing algorithms simply due to case differences, thereby reducing the accuracy of results. This standardisation helps create a uniform dataset, which is crucial for accurate analysis. To further refine the dataset, the contractions in the text data is also expanded; for example, “don’t” is converted to “do not.” This ensures that words are not counted separately, thus maintaining consistency.

**Fig 6:**
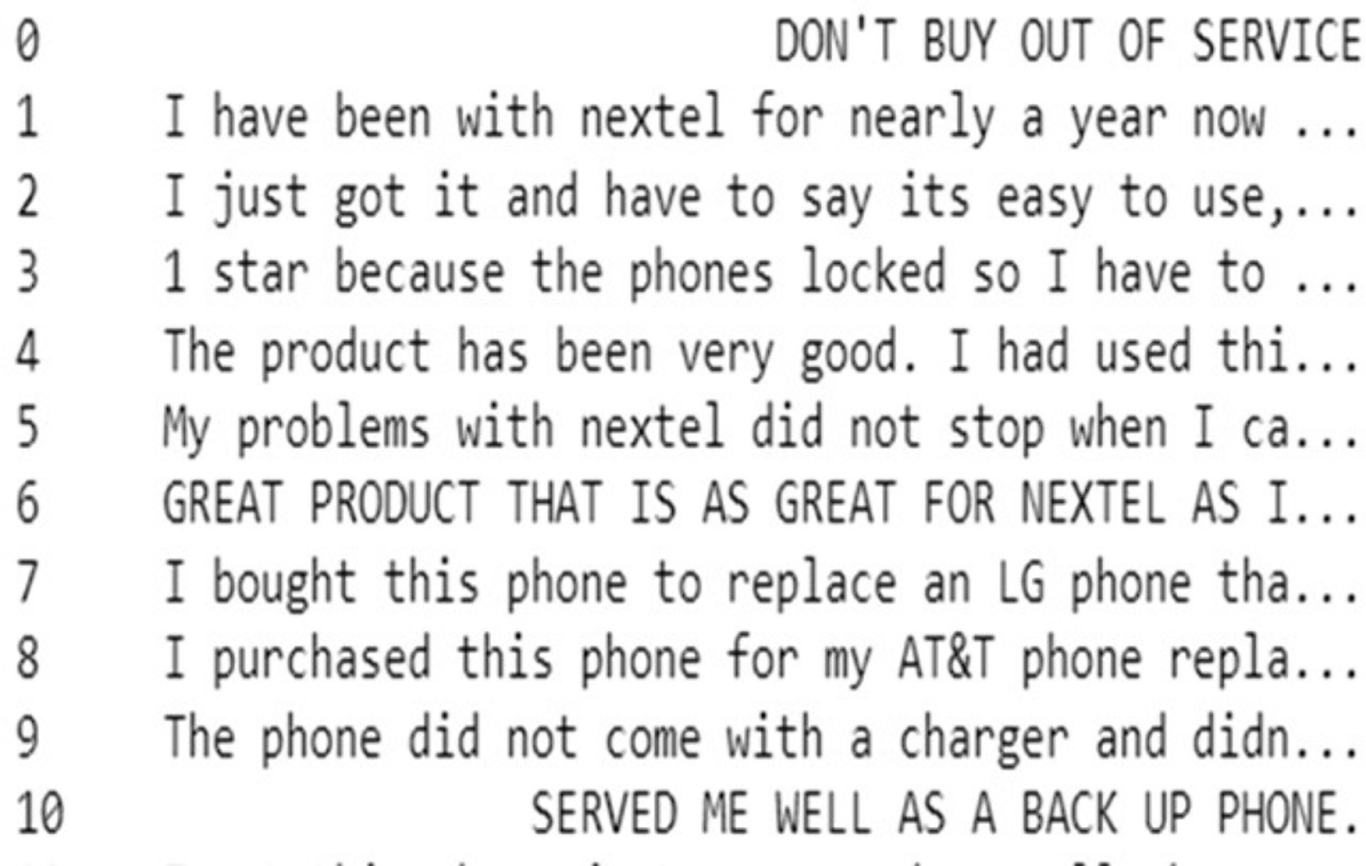
Original Text Data.

**Fig 7:**
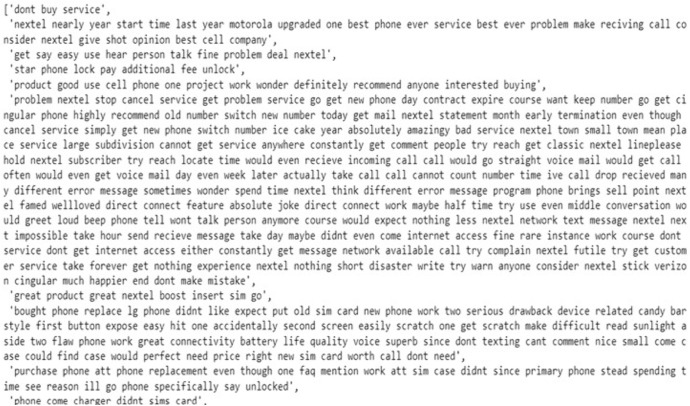
Final Processed Text Data.

After that, Natural Language Toolkit (NLTK)‘s predefined stop-words list is used to remove common stop words like “a,” “the,” and “is.” This enhances model performance by highlighting important words that carry the meaning of the text. In the last step of our text preprocessing, lemmatization with POS tagging is applied by using WordNet Lemmatizer from NLTK. POS tagging involves assigning a specific label such as noun, verb, adjective, etc, to each word in the sentence. This labelling is essential because it provides contextual information that helps in accurate lemmatization. Lemmatization helps to reduce words to their base form and standardise text data. For instance, the word “purchasing” is reduced to its base form “purchase.” By applying the lemmatization process, the text data was further cleaned and standardized, reducing words to their root forms while preserving their essential meanings. This comprehensive preprocessing ensures our dataset is well-prepared for subsequent sentiment analysis shown in Fig 6 and Fig 7.

Following these preprocessing steps, the TF-IDF (Term Frequency-Inverse Document Frequency) method is employed for feature extraction. TF-IDF is calculated using the formula [15], which is illustrated in Equation 1:


TF−IDF(t,D,d)=TF(t,d)×IDF(t,D)       (1)

The TF-IDF scheme is particularly effective at emphasizing terms that frequently appear in each document. Term frequency (TF) quantifies the frequency of a term’s occurrence within a specific document. The TF-IDF method combines (Term Frequency) TF with the inverse document frequency (IDF) to highlight the significance of a term within a document relative to the entire dataset.

The pre-processed text data is then converted into numerical features that can be used for ML models. The TfidfVectorizer not only computes the Term Frequency-Inverse Document Frequency (TF-IDF) values but also manages tokenization internally. These preprocessing and feature extraction techniques are essential for transforming raw text data into a format that enhances the effectiveness of subsequent analysis. Finally, the pre-processed text data was combined with essential attributes, such as “brand,” “rating,” and “sentiment,” for subsequent sentiment analysis.

### 3.5. Data splitting

To prepare the data for model training and prediction with traditional classification models, data splitting was performed using the train_test_split function. This step divided the pre-processed dataset into training and testing subsets to ensure that the ML models could be effectively trained and evaluated. Specifically, 80% of the data was allocated to the training set, while 20% was reserved for the testing set. The testing set serves as a separate subset to evaluate the model’s performance and generalizability on unseen data. This split is crucial for assessing how well the model performs.

On the other hand, DL models often work directly with raw or minimally processed data. Sentiment labels were converted into numerical format and then one-hot encoded using LabelEncoder. Texts were tokenized with the Tokenizer, configured to manage up to 10,000 words and out-of-vocabulary tokens. The tokenized sequences were then padded to a uniform length of 100 tokens. Subsequently, train_test_split was used again to divide the data into 80% of training and 20% of testing subsets. This ensured that both types of models received appropriately formatted and split datasets for effective training and evaluation.

### 3.6. Hyperparameter tuning strategy

To ensure fair and systematic optimisation across models, a grid-search procedure was applied to both traditional ML and DL algorithms. For Decision Trees, the grid included max_depth ∈ {200, 400, 600, 800, 900}. Naïve Bayes was tuned with alpha ∈ {1.0, 0.5, 0.1, 0.05}. Logistic Regression explored C ∈ {0.1, 1.0, 10}, solver ∈ {lbfgs, saga, sag}, and max_iter ∈ {100, 200, 300}. The SVM grid covered C ∈ {1, 5, 10}, kernel ∈ {linear, rbf}, and gamma ∈ {scale, auto}.

For DL models, the CNN grid comprised kernel_size ∈ {3, 5, 7}, filters ∈ {64, 128}, and dropout_rate ∈ {0.2, 0.5}; training epochs were capped at 20 with Early Stopping patience of 3. The RNN and LSTM grids varied units ∈ {64, 100}, dropout ∈ {0.2, 0.3}, and learning_rate ∈ {0.001, 0.0005}. All grids were evaluated with five-fold cross-validation on the training set, and the best hyperparameter combination for each algorithm was selected according to mean validation accuracy. These chosen settings form the basis of the fine-tuning results reported in Section 4.

### 3.7. Identified models

#### 3.7.1. Traditional machine learning models.

The selection of traditional ML models for this study was guided by their established performance and interpretability in text classification and sentiment analysis tasks. Tree-based model such as Decision Trees was included because of their ability to handle nonlinear relationships, mixed feature types, and feature importance estimation. Naive Bayes models were chosen for their probabilistic foundation and efficiency in high-dimensional text data, particularly where word independence assumptions hold approximately true. Logistic Regression models were selected for their robustness, ease of interpretation, and effectiveness as strong linear baselines in sentiment prediction problems. SVM models were included due to their proven capability to generalise well in sparse feature spaces and to achieve strong discriminative boundaries in text-based classification. The models were assessed based on accuracy, F1 score, precision, recall. Neutral sentiments may be underrepresented in the dataset compared to positive and negative reviews. This imbalance can lead the model to focus more on the majority classes and perform poorly on the minority class. If the features of neutral reviews are not distinct enough, the model may struggle to classify them accurately.

Other ML were not considered in order to maintain methodological focus and computational efficiency, as the primary aim was to benchmark the performance of diverse learning paradigms (probabilistic, linear, and non-linear) against advanced DL models. The selected models therefore represent a balanced mix of interpretability, generalisability, and computational practicality, making them well-suited to the sentiment classification objectives of this study.

**3.*7*.1.1 Decision Trees:** Decision Trees model has a tree-like structure, where each node represents a decision or test on an attribute, branches indicate the possible outcomes of these decisions, and leaf nodes signify the final predictions. In the context of classifying customer reviews, a hierarchy of decision nodes is built based on review features to categorise the reviews into different sentiment categories. In the context of our dataset, entropy is a key concept used to measure the uncertainty or randomness in the data, specifically in how the data is classified into different sentiment categories. The formula of splitting criterion – Entropy from [31] is shown below in Equation 2.


$$Entropy\;(S)=\;\sum_{c\;\in C}-\;p(c){log}_{\text{2}}p(c)\;$$ (2)

where S is the dataset that entropy is calculated, c is the sentiment classes in set S, and p(c) – the proportion of elements belonging to class c in the dataset S.

**3.7.1.2 Naive Bayes Model:** Naive Bayes is a probabilistic approach for text classification that operates under the assumption that documents are created by a distribution determined by unseen parameters. The model estimates these parameters from the training data and uses Bayes’ theorem to calculate the likelihood of each class. It classifies documents by selecting the class with the highest probability. Naive Bayes model would first calculate the probability of the class [31], which is illustrated as in Equation 3:


$$P\left(c|x\right)=\;\frac{P\left(x|c\right)P(c)}{P(x)}\;$$ (3)

Here c represents data belonging to a particular sentiment class, x is the data with an unknown class, P(c|x) represents the posterior probability of the class given the predictor, P(x|c) is the predictor probability of the given class, P(c) – prior probability of the class, P(x) is the prior probability of the predictor.

**3.7.1.3 Logistic Regression Model:** Logistic Regression is a supervised learning technique that predicts the probability of a target variable by modelling the likelihood of a data point belonging to a specific class [32]. This model utilizes the logistic function, which maps input values to a probability range between 0 and 1, allowing it to classify data into discrete categories. The formula of Logistic Regression model [33] is illustrated as in Equation 4:


$$S(x)=\;\frac{\text{1}}{\text{1}+\;e^{-x}}=\;\frac{e^{x}}{e^{x}+\text{1}}\;$$ (4)

where S(x) represents logistic function or sigmoid function, p refers to the probability of the dependent variable belonging to a particular class, e is the exponential function, and x means linear combination of the input features. This method is applicable in both binary and multiclass classification scenarios. According to the dataset used for this study, this model will estimate whether a review belongs to a positive, negative, or neutral category, and assigns it to the most likely category.

In this study, Logistic Regression was implemented using a multinomial (SoftMax) formulation to accommodate the three sentiment categories (positive, negative, and neutral). The model was trained using the LogisticRegression class in scikit-learn with the parameter setting multi_class = ‘multinomial’ and the solver lbfgs. This approach estimates a separate set of coefficients for each sentiment class relative to a common baseline and computes class probabilities through the SoftMax function. The predicted sentiment label is then assigned based on the class with the highest probability. This configuration ensures that the model captures the full range of sentiment categories within a unified probabilistic framework, providing interpretable and generalisable classification outputs.

**3.*7*.1.4 Support Vector Machine Model:** Support Vector Classifier (SVC) is a powerful supervised ML algorithm that is widely used in classification tasks. This model operates by identifying the optimal hyperplane that best separates different classes within a multidimensional space. The goal of the SVC is not just to find any hyperplane but to discover the one that maximizes the margin between classes, ensuring that the separation is as clear and distinct as possible [34,35]. The SVM model formula [14] is presented as follows in Equation 5:


$$W^{T}X-b=\text{0}\;$$ (5)

where W represents the weight of vector perpendicular to the hyperplane, X is the feature vector of a data point, and b is the bias term that adjusts the hyperplane from the origin. The margin is the distance between hyperplane and the closest data points from each class. The larger the margin, the better the model performance [32]. This combination of mathematical precision and strategic placement of the hyperplane makes SVC a highly effective tool in the realm of ML, particularly for tasks involving complex and high-dimensional data.

**3.7.1.5 Training Procedure for Machine Learning Models:** To ensure consistent evaluation across all ML classifiers, a standardized training procedure was adopted. Each model, Decision Trees, Naive Bayes, Logistic Regression, and SVM was implemented using the scikit-learn library in Python (version 1.2.2). Prior to training, the dataset was split into 80 percent training and 20 percent testing subsets using stratified sampling, as described in Section 3.5. Feature vectors were extracted using TF-IDF vectorization, preserving token frequency information critical for classification. Hyperparameters for each model were optimized through grid search with five-fold cross-validation, using validation accuracy as the scoring metric. For Decision Trees, the maximum depth was varied in the range of 200–900; Logistic Regression was optimized across different solvers (lbfgs, saga, sag) and regularization strengths (C = 0.1 to 10); SVM tuning involved kernel choice (linear, RBF), penalty parameter (C = 1–10), and gamma settings. Naive Bayes used smoothing values (alpha) between 0.05 and 1.0. Class weights were adjusted in all models where applicable to handle the sentiment imbalance described in Section 3.2. All models were trained and tested under the same random seed (42) to enable reproducibility. Final model performance was reported based on the test set using metrics described in Section 3.7.3.

#### 3.7.2. Deep learning models.

In addition to the traditional ML models, DL models were also explored in this study to provide a broader comparison. These models known for their ability to capture complex patterns in data were included to assess whether they could outperform traditional models. In this study, deep neural network models including CNN, RNN and long-short term memory (LSTM) classifiers were employed.

**Fig 8:**
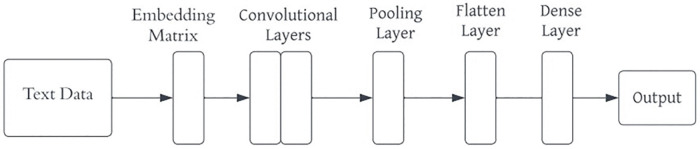
Architecture of Convolutional Neural Network Model.

**3.7.2.1 Convolutional Neural Network:** CNN are powerful feedforward neural networks commonly applied in fields like image classification or object recognition. CNN have recently been adapted for text classification with impressive results due to their ability to automatically learn and extract meaningful features from raw data. A simple architecture of CNN is shown as Fig 8 below.

CNN consists of embedding layer, convolutional layer, pooling layer, flatten and dense layer. The process begins with an embedding layer, which converts raw text into numerical representations since ML and DL algorithms cannot process raw text directly. The convolutional layer identifies high-level features through a convolution operation by sliding the filters over the input sequence. After this, the pooling layer reduces the dimensionality of the feature maps to decrease computational complexity and highlight the most relevant features [16]. Sometimes, the flatten layer is used to convert the feature maps into a single column vector, making them suitable for input into the dense layer. The dense layer is then employed to determine the class label, utilizing SoftMax activation functions for tasks involving multi-class classification.

In this study, this model begins with an embedding layer that is added to convert word indices into dense vectors of fixed size and followed by a Conv1D layer with 128 filters and a kernel size of 5. ReLU activation function is then applied to capture local features from the text. Next, a MaxPooling1D layer with a pool size of 2 is used to retain the most important features. A Dropout layer with a dropout rate of 0.5 is included to prevent overfitting. Following this, a GlobalMaxPooling1D layer further down-samples the input representation by taking the maximum value across the entire dimension to reduce the computation while retaining crucial features. The model then includes a Dense layer with 128 units and a ReLU activation function for additional processing, followed by another Dropout layer to further mitigate overfitting. The final layer is a Dense layer with 3 units, corresponding to the three sentiment classes, and a SoftMax activation function to output a probability distribution over the classes. The model is compiled using the categorical cross-entropy loss function, which is appropriate for multi-class classification problems, and the Adam optimizer, known for its efficiency and adaptive learning capabilities.

The CNN was trained for 20 epochs using a batch size of 32. These parameters were selected through preliminary experiments involving a manual grid search approach. The kernel size of 5 was chosen based on its ability to capture short-range dependencies in review text, offering a balance between local pattern extraction and computational efficiency. Early experimentation showed that kernel sizes smaller than 5 underperformed in validation accuracy, while larger sizes increased training time without measurable gains. Similarly, the choice of 20 epochs was based on early stopping criteria and validation curve trends. The model exhibited peak performance around Epoch 15 and began showing signs of overfitting beyond that point. Training beyond 20 epochs offered minimal additional benefit while increasing the risk of overfitting and computational cost. These observations guided the final hyperparameter configuration, which is further discussed in the Results and Discussion sections.

**3.7.2.2 Recurrent Neural Network:** A RNN is a specialized form of artificial neural network designed to process sequential data, making it particularly effective for tasks such as language translation, NLP, and speech recognition [17]. RNN have a unique ability to retain information from previous inputs, which allows them to influence current and future outputs, enhancing their learning capability from training data [16]

The formula for RNN model is shown as in Equation 6:


$$h_{t}=\text{tanh}\left(b+W_{ht-\text{1}}+U_{xt}\right)\;$$ (6)

where b is bias value, $W_{ht-\text{1}}$ is the previous hidden state, which carries information from past time steps and $U_{xt}$ is current input, which determines its influence on the current hidden state. This can be seen as a process where the RNN updates its memory (hidden state) at each time step by combining information from the previous hidden state and the current input, passing this combination through a non-linear activation function (tanh). This allows the network to process sequences of data in a way that invovle both the current input and the history of past inputs.

**Fig 9:**
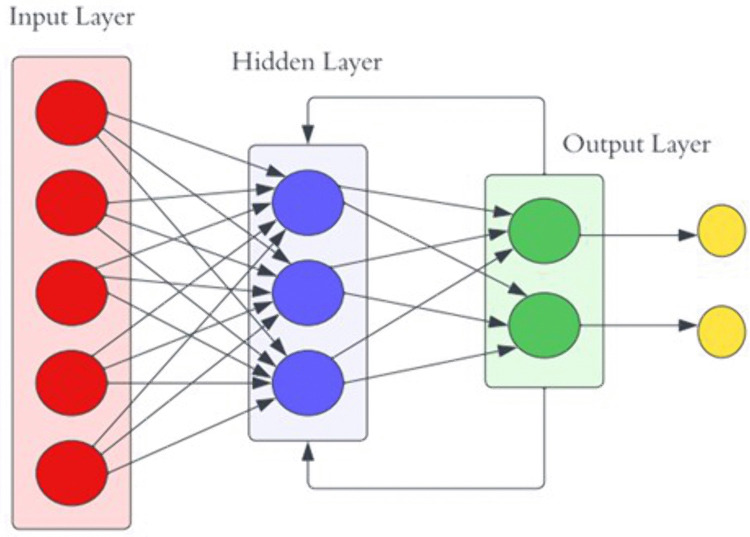
Architecture of Recurrent Neural Network Model.

The RNN model shown in Fig 9 in this study is designed with an Embedding layer, followed by a SpatialDropout1D layer with a dropout rate of 0.2 to reduce overfitting. A single LSTM layer with 100 units is used with both dropout and recurrent dropout rates of 0.2 to further prevent overfitting. Adding LSTM layer in a model helps capture more complex patterns and relationships in the data. The model also includes a Dense layer with 128 units and ReLU activation to capture complex features, and a final Dense layer with SoftMax activation for three-class sentiment classification.

**Fig 10:**
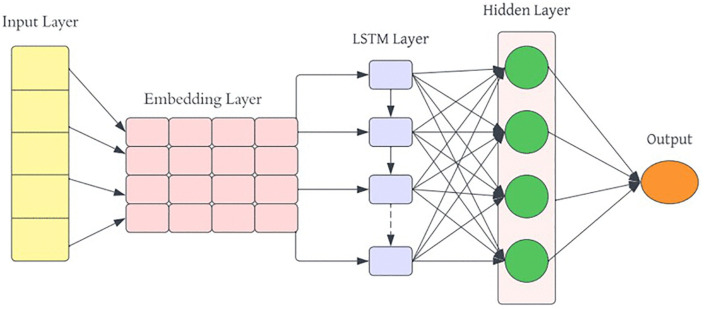
Architecture of Long Short-Term Memory Model.

**3.7.2.3 Long Short-Term Memory:** LSTM is a specialized form of RNN designed to capture and learn long-term dependencies in data [20]. The architecture of LSTM is outlined in Fig 10. In LSTM model, the pre-processed input data is represented as an embedding matrix. The LSTM layers with number of units will process the data, followed by fully connected layer for classification task. The final layer will use an activation function to reduce the dimensional input vector down to a single output.

**Fig 11:**
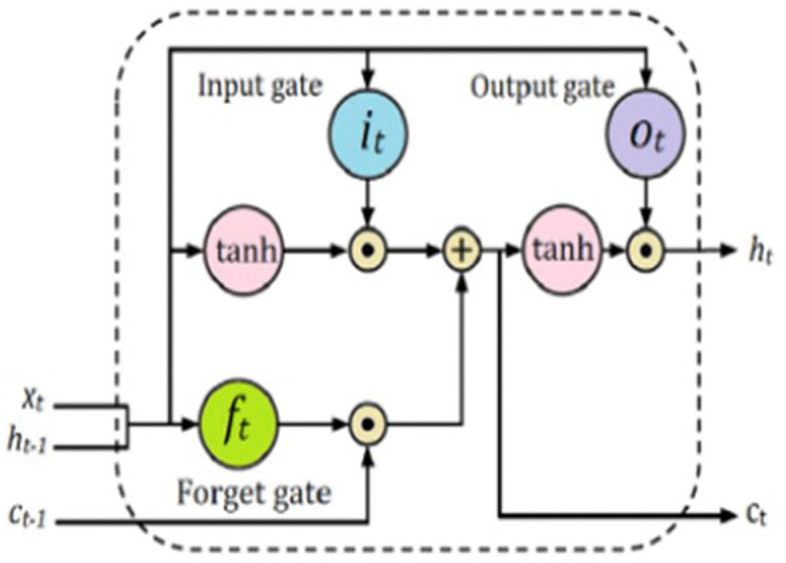
Mechanism of Long Short-Term Memory Model.

Moreover, Fig 11 shows the mechanism of LSTM model. LSTM consists of a memory cell (ct) and three key non-linear gates: the input gate (it), the forget gate (ft) and the output gate (ot). The memory cells maintain a consistent state over time, while the gates control the flow of information into and out of the cell to ensure that relevant data is retained or discarded as needed. These mechanisms allow LSTMs to effectively retain relevant data over time. In short, LSTM networks employ a memory cell along with a set of gates to manage the storage, updating, and retrieval of information across extended sequences. These mechanisms allow LSTMs to effectively retain relevant data over time [17].

In this study, the LSTM model was constructed starting with an Embedding layer, followed by a SpatialDropout1D layer, like the architecture used in the CNN and RNN models. This model consists of three LSTM layers, each with 100 units and dropout settings of 0.3 for both standard dropout and recurrent dropout. These layers enable the model to learn complex temporal relationships within the data. After the LSTM layers, there is a Dense layer with 128 units and ReLU activation for additional feature processing. To avoid overfitting, a Dropout layer with a 0.5 rate is included. The final output is a Dense layer with 3 units and a SoftMax activation, suited for multi-class sentiment classification. Additionally, Reduce Learning Rate On Plateau (ReduceLROnPlateau) is used to lower the learning rate of the model when validation accuracy has stopped improving.

**3.7.2.4 Training Procedure for Deep Learning Models:** All DL experiments followed a unified training protocol to support reproducibility. For every network, the dataset was shuffled and stratified into 80 percent training and 20 percent testing splits as described in Section 3.5. Models were built with the Keras implementation in TensorFlow 2.14 and trained on a single NVIDIA RTX 3060 GPU. The Adam optimizer was used with an initial learning_rate of 0.001 for the CNN and RNN, and 0.0005 for the LSTM network, reflecting the grid selections in Section 3.6. The batch_size was fixed at 32 across models for comparability. Early Stopping monitored validation loss with patience = 3 and restored the best performing weights. A ReduceLROnPlateau callback halved the learning rate if validation accuracy plateaued for two epochs. Each model was permitted a maximum of 20 epochs, although Early Stopping typically halted training earlier (CNN at epoch 7, RNN at epoch 9, LSTM at epoch 16). Training history, including loss and accuracy per epoch, was logged and the final weights were saved with a version tag corresponding to the best validation epoch. The same random seed (42) was set for NumPy and TensorFlow to ensure repeatability of results.

#### 3.7.3. Evaluation metrics.

To compare the effectiveness of various models, multiple evaluation metrics including accuracy, precision, recall, and F1-score are employed. By analysing these metrics, strengths and weaknesses across different models can be identified, allowing for the selection of the most effective model for the task. Each metric offers insights into how well the models classify data.

- **Accuracy:** Accuracy is a metric that measures the proportion of correct predictions made by a classification model out of the total number of predictions. It reflects how well the model’s predictions align with the actual values as shown in Equation 7:

$$\text{AC}=\;\frac{number\;of\;correct\;predictions}{Total\;number\;of\;predictions}\;$$ (7)

- **Precision:** This metric evaluates the proportion of relevant items among those selected by the model as shown in Equation 8:

$$\text{P}=\;\frac{\text{TP }}{\text{TP}+\text{FP}}\;$$ (8)

where P refers to precision, TP is the True Positive, which the instances where the model correctly identifies the positive class, and FP is False Positive which the instances where the model incorrectly predicts a positive class.

- **Recall:** This metric assesses the proportion of relevant items that the model successfully identifies as shown in Equation 9:

$$\text{R}=\;\frac{\text{TP }}{\text{TP}+\text{FN}}\;$$ (9)

where, FN is False Negative. The instances where the model fails to identify a positive class and incorrectly predicts it as negative.

- **F1-Score**: This metric provides a balance between precision and recall. It ranges from 0 (indicating poor performance) to 1 (indicating excellent performance) as shown in Equation 10:

$$\text{F}{\text{1}}_{\text{Score}}=\;\frac{\text{2}\text{ x P x R }}{\text{P}+\text{R}}\;$$ (10)

During evaluation, cross validation was utilized to evaluate the effectiveness of the models. Cross validation is a technique used to assess the performance of a model by evaluating its ability to make accurate predictions on new data [14]. The models were cross validated 5 times to ensure robust evaluation in this study. All model evaluations, including those during hyperparameter tuning and final testing, were conducted using five-fold cross-validation with stratified sampling to preserve class distribution across folds. Random shuffling of the dataset was performed before splitting to ensure robustness and reduce ordering bias. The same random seed (42) was used across all experiments to support reproducibility.

### 3.8. Brand topic detection model

To determine the topics in this dataset, two topics modelling are used in this section. Topic modelling can also be used for a variety of tasks such as detecting trends on social media or uncovering hidden themes in large text datasets. In this study, three topic detection models including LDA, and NMF are used to reveal insights from the dataset.

#### 3.8.1. Latent Dirichlet allocation.

**Fig 12:**
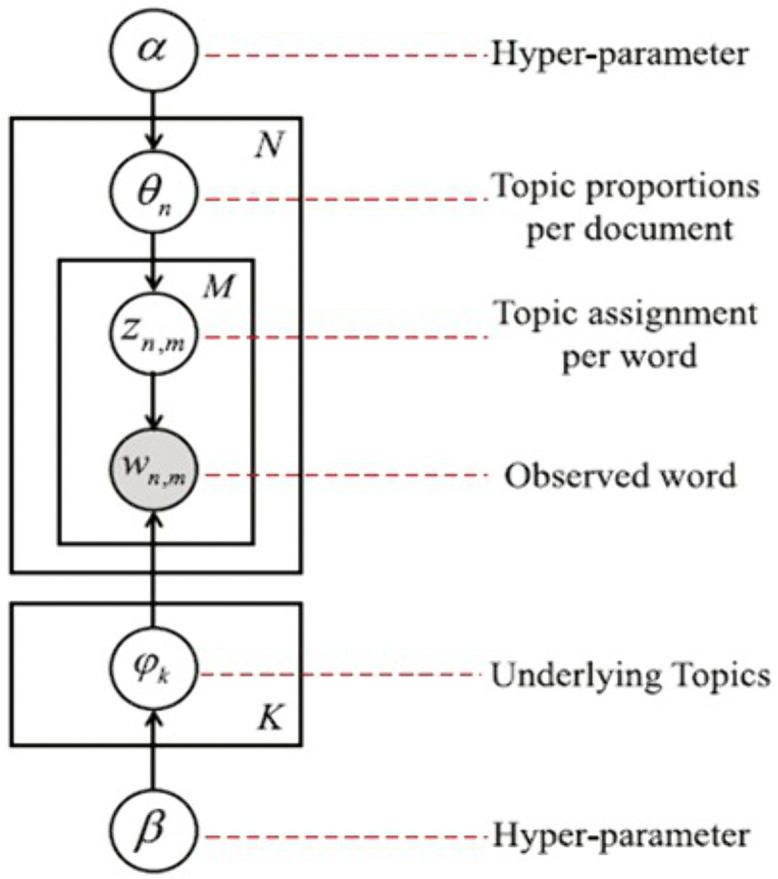
LDA Model.

LDA model is a generative probabilistic model that assumes K latent topics are hidden in given N documents corpus. This method is particularly useful in revealing the thematic structure of the dataset and allows for a nuanced understanding of the dominant topics present in the customer reviews. The generative process for each document in the text archive is presented as Fig 12 below.

This process outlines how each document is generated in the entire corpus. Each document in the corpus is assumed to be a mix of these topics, and the words within the document are drawn from this mixture. The generative process is repeated for each document, leading to a probability model for the entire corpus. The model’s hidden variables, which represent the topic-word distributions (ϕ) and document-topic distributions (*θ*), are estimated using techniques like Gibbs sampling and variational algorithms by maximizing the probability P(D∣α, β) of the observed data given the model parameters. The formula of probability [22] is shown in Equation 11:


$$P\left(D|\alpha ,\beta \right)=\;\prod_{n=\text{1}}^{N}\int P\left(\theta_{n}|\alpha \right)F(\theta ,\varphi )d\theta_{n}\;$$ (11)

The equation represents the likelihood of observing the entire corpus 𝐷, given the model's hyperparameters 𝛼 and 𝛽. The probability is denoted as 𝑃 (𝐷∣𝛼, 𝛽), where 𝛼 controls the distribution of topics within documents (document-topic distribution) and 𝛽 controls the distribution of words within topics (topic-word distribution). $\prod_{n=\text{1}}^{N}\;$indicates that the likelihood is calculated as a product over all documents in the corpus (where 𝑁 is the total number of documents). The function 𝐹 (𝜃, 𝜙) represents the probability of observing all the words in document given the topic distribution and the topic-word distributions. In short, the process involves summing over all topic assignments for each word in each document and then multiplying these probabilities across all words and documents.

The LDA model was constructed to uncover the five main topics within customer reviews across various brands. The text data was pre-processed and converted into a bag-of-words format. Using this corpus, an LDA model was constructed with a predefined number of topics, K as five topics. The model assumes that each review is a mixture of these topics, with each topic represented as a distribution over words. The final topics, represented by the top words within each topic, provide insights into the main themes filtered by each brand in customer reviews.

#### 3.8.2. Non-negative matrix factorization.

NMF is a technique used to simplify complex data by breaking it down into non-negative components. NMF helps uncover hidden topics within the document by expressing each document as a combination of a few key themes. For each document, NMF represents it as a sum of these components, with specific weights showing how much each component contributes to that document.

The process of NMF finding the best combination of components is done by minimizing the difference between the original data and the reconstructed version based on these components. The goal is to approximate the original matrix 𝐷 (which contains the data) as the product of two non-negative matrices, U and V. This is represented by the equation [22] below in Equation 12:


$$\begin{array}{c} min\\ U,V \end{array}\;\left||D-UV|\right|\begin{array}{c} \text{2}\\ F \end{array}\;$$ (12)

where D is the original data matrix, U represents the topics, V shows how these components mix to recreate the original data and ∥ ⋅ ∥ F denotes the Frobenius norm, which measures the difference between the original and approximated matrices.

Moreover, the formulas for U and V [22] are shown as below in Equation 13 and equation 14:


$$U\;\leftarrow U\frac{DV^{T}}{UVV^{T}}$$ (13)

$$V\leftarrow V\frac{U^{T}D}{U^{T}UV}\;$$ (14)

These equations adjust U and V to minimize the difference between the original data and its approximation. The optimization process can be done by adjusting U to better fit the data D based on how well the current V represents D or adjusting V to better fit the data D based on how well the current U represents D.

In this study, an NMF model was built to uncover five topics by brand. The process began by transforming the text data into a numerical format using Term Frequency-Inverse Document Frequency (TF-IDF) vectorization. This method captures the importance of words in each document while considering their frequency across the entire corpus. The top ten words associated with each topic were then extracted to provide insights into the primary themes present in the reviews.

#### 3.8.3. Topic coherence evaluation.

To evaluate the quality of topics generated by the LDA and NMF models, we calculated the Coherence Score (CS) using the c_v metric. The CS measures the degree of semantic similarity between the most relevant words within a topic, thereby reflecting its interpretability. A higher CS suggests that the top words in a topic are more meaningfully related and thus more easily understood by humans. This metric is widely used in topic modelling evaluations to provide a quantitative assessment of how coherent or useful the generated topics are. In this study, CS values were computed using the gensim library in Python.

### 3.9. Brand sentiments

After identifying the topics, the VADER sentiment analyser was applied to assess the sentiment associated with each brand. VADER is well known at analysing the sentiment of texts, including those with emojis and other social media-specific elements that other libraries might overlook. VADER provides four different sentiment scores: positive, negative, neutral, and compound. The positive, negative, and neutral scores represent the proportion of the text that falls into each category, while the compound score is a combined metric that sums up the overall sentiment of the text. This compound score ranges from −1 (indicating extremely negative sentiment) to 1 (indicating very positive sentiment) [4]. In this study, only the compound score is used to assess sentiment.

**Fig 13:**
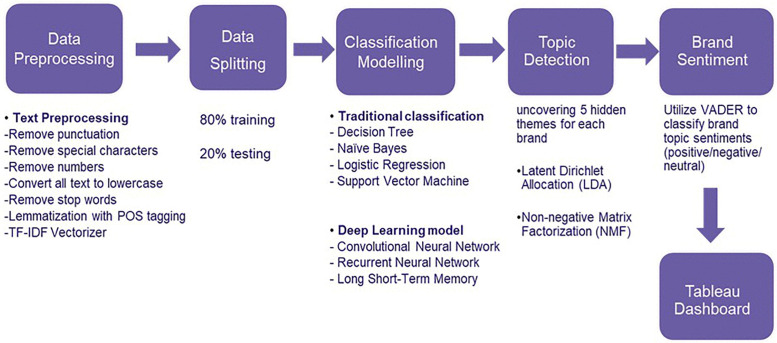
Proposed Model.

By linking the sentiment scores to the topics generated by the topic detection model, the average sentiment for each topic across all reviews was calculated. This approach provided us with insights into not only the key themes present in the customer feedback but also the overall sentiment associated with each theme. For example, a topic related to phone camera feature might have a positive sentiment, while another topic related to product quality might be neutral. The results were categorized into sentiment types, Positive, Neutral, and Negative, based on the average sentiment scores, allowing for a clear understanding of how customers feel about various aspects of the brands they reviewed. Brand sentiment analysis is crucial for businesses to understand how customers perceive their products and services. By evaluating sentiments, companies can gain valuable insights into customer satisfaction, identify areas for improvement, and tailor their marketing strategies effectively. Overall, this model shown in Fig 13 allowed us to effectively combine topic modelling and sentiment analysis to gain a deeper understanding of customer opinions for each brand.

## 4. Results & Discussion

In this section, the results from both traditional ML models and DL techniques are examined. The outcomes of each model are presented, with their performances compared to determine the most effective approach for the dataset. Additionally, the results of the brand topic detection model are explored, and each method is evaluated to identify the most suitable model. Finally, the VADER sentiment analysis tool is applied to assess the sentiment scores associated with the identified topics.

### 4.1. Performance analysis on traditional machine learning models

#### 4.1.1. Decision trees model.

As detailed in Table 3, the Decision Trees model achieved an average accuracy of 77.29% after running five cross-validation. It performs particularly well on the majority class, which is positive sentiments, with an average precision, recall, and F1 score of 0.87. In contrast, its performance on negative sentiments is less robust, with average metrics of 0.66. This indicates that the model is better at identifying and classifying positive reviews compared to negative ones. For neutral sentiments, the model struggles to recognize them, achieving only an F1-score of 0.20. The tree’s depth of 937 suggests that the model is quite complex, involving multiple decision nodes to classify the data.

**Table 3:** Decision Trees Model Performance.

Depth of Decision Trees = 937			
Metrics	Negative	Neutral	Positive
Support	3261	941	9350
Precision	0.66	0.23	0.86
Recall	0.66	0.17	0.88
F1-Score	0.66	0.20	0.87
Cross-Validation Score (cv = 5)	0.7730, 0.7737, 0.7722, 0.7723, 0.7734		
Mean Cross-Validation Accuracy	0.7729		

To improve the performance of the Decision Trees model, fine-tuning was conducted by adjusting the key parameter, max_depth, to explore different values. Max Depth refers to the maximum number of splits allowed in a Decision Trees and determines how deep the tree can grow, with each level representing a decision based on a feature in the dataset. During fine-tuning, it was observed that the model’s performance did not improve significantly as demonstrated in Table 4 and Table 5. After running cross-validation for each max_depth value, a maximum depth of 600 yielded an average accuracy of 77.31%.

**Table 4:** Accuracy of Decision Trees Model Performance with Different Max_Depth After Running Five Cross-Validation.

Decision Trees Model Max Depth	600	700	800	900
Cross-Validation Score (cv = 5)	0.7714	0.7733	0.7730	0.7730
	0.7761	0.7733	0.7737	0.7734
	0.7721	0.7722	0.7722	0.7722
	0.7747	0.7723	0.7723	0.7723
	0.7705	0.7713	0.7735	0.7735
Mean Cross-Validation Accuracy	0.7731 (Highest Accuracy)	0.7729	0.7725	0.7729

**Table 5:** Decision Trees Model Performance After Fine-Tuning.

Depth of Final Decision Trees = 600			
Metrics	Negative	Neutral	Positive
Support	3261	941	9350
Precision	0.66	0.22	0.86
Recall	0.66	0.18	0.87
F1-Score	0.66	0.20	0.87
Mean Cross-Validation Accuracy	0.7731		

#### 4.1.2. Naive Bayes model.

The Naive Bayes model is known for its high speed and efficiency in text classification tasks. As shown in Table 6, it achieved an average accuracy of 78.01%, which is comparable to the Decision Trees model’s performance. The Naive Bayes model outperforms the Decision Trees model in terms of recall for positive sentiments, achieving a high recall rate of 0.99. This means that Naive Bayes is highly effective at identifying positive reviews. However, its precision is lower than that of the Decision Trees model, standing at 0.78. This indicates that while Naive Bayes is particularly good at detecting positive sentiments, it is less accurate in ensuring that all detected positive reviews are genuinely positive. Conversely, the Naive Bayes model struggles significantly with neutral sentiments, scoring zero across all evaluation metrics for this category. This suggests that the model fails to identify or predict neutral reviews, which might be attributed to factors such as dataset imbalance, insufficient representation of neutral sentiments, or the model’s inability to distinguish neutral reviews effectively. On the other hand, the model performs well with negative sentiments, showing a high precision of 0.88, meaning it correctly identifies negative reviews 88% of the time. Nonetheless, its recall for negative sentiments is only 0.47, indicating that it captures just 47% of all actual negative reviews.

**Table 6:** Naive Bayes Model Performance.

Naïve Bayes Model			
Metrics	Negative	Neutral	Positive
Support	3261	941	9350
Precision	0.88	0.00	0.78
Recall	0.47	0.00	0.99
F1-Score	0.61	0.00	0.87
Cross-Validation Scores (cv = 5)	0.7802, 0.7799, 0.7804, 0.7821, 0.7779		
Mean Cross-Validation Accuracy	0.7801		

To further enhance the performance of the Naive Bayes model, fine-tuning was performed by adjusting the key parameter, alpha. In Naive Bayes models, alpha is a smoothing parameter used to handle zero probabilities in the estimation of probabilities for unseen words in the training data. After fine-tuning, the model’s accuracy improved to 82.69%, outperforming the previous version with a smaller alpha value of 0.05. With a smaller alpha, the model is less likely to assume that all words occur with a uniform probability. As a result, the recall for negative sentiments improved significantly from 0.47 to 0.71, indicating a better ability to correctly identify negative reviews. Additionally, the F1 score for neutral sentiments also saw a slight improvement, reflecting some progress in predicting neutral reviews. Table 7 presents the results of the Naive Bayes model after fine-tuning.

**Table 7:** Naive Bayes Model Performance After Fine-Tuning.

Naïve Bayes Model (alpha = 0.05)			
Metrics	Negative	Neutral	Positive
Support	3261	941	9350
Precision	0.78	0.24	0.86
Recall	0.71	0.04	0.96
F1-Score	0.74	0.07	0.90
Cross-Validation Scores (cv = 5)	0.8228, 0.8270, 0.8267, 0.8283, 0.8298		
Mean Cross-Validation Accuracy	0.8269		

#### 4.1.3. Logistic regression.

For classification tasks, logistic regression is a supervised ML algorithm designed to predict the likelihood that a given instance belongs to a specific class. The logistic regression model achieved an accuracy of 85.05%, making it the best-performing model so far. Based on Table 8, it performs very well on positive sentiments, with f1-score of 0.92, and 0.78 on negative sentiments. Nonetheless, the model struggles with neutral sentiments, achieving only 26% precision, 2% recall, and a 4% F1-score. This low performance could be due to the limited number of neutral sentiment instances, meaning the model may not have enough examples to learn from. Additionally, logistic regression might be better at distinguishing between obvious different classes (positive vs. negative) and struggle with the minor distinctions required for neutral sentiments.

**Table 8:** Logistic Regression Model Performance.

Logistic Regression Model (Regularization Strength C = 1.0, Number of Iterations = 205, Solver used = lbfgs)			
Metrics	Negative	Neutral	Positive
Support	3261	941	9350
Precision	0.77	0.26	0.89
Recall	0.80	0.02	0.95
F1-Score	0.78	0.04	0.92
Cross-Validation Scores (cv = 5)	0.8456, 0.8500, 0.8511, 0.8527, 0.8530		
Mean Cross-Validation Accuracy	0.8505		

To improve the performance of the logistic regression model, fine-tuning was conducted by adjusting several parameters. Specifically, a range of values for the regularization strength parameter C was explored, which governs the trade-off between achieving a good fit on the training data and maintaining smaller model coefficients. Additionally, different solvers (sag, lbfgs, and saga) were assessed to determine the optimization algorithm used, and various settings for the max_iter parameter were examined, which specifies the maximum number of iterations allowed for the solver to converge. Although these adjustments were made, the enhanced logistic regression model did not improve significantly with accuracy of 85.07% as shown in Table 9.

**Table 9:** Logistic Regression Model Performance After Fine-Tuning.

Best Logistic Regression Model (Regularization Strength C = 1.0, Number of Iterations = 100, Solver used = lbfgs)			
Metrics	Negative	Neutral	Positive
Support	3261	941	9350
Precision	0.77	0.28	0.89
Recall	0.80	0.03	0.95
F1-Score	0.78	0.05	0.92
Cross-Validation Scores (cv = 5)	0.8464, 0.8500, 0.8514, 0.8531, 0.8524		
Mean Cross-Validation Accuracy	0.8507		

#### 4.1.4. Support vector machine.

Support Vector Machines (SVMs) are a type of supervised ML algorithm that works by finding the optimal boundary that best separates different classes. Table 10 and Table 11 present the detailed results of the SVM model, including its performance after fine-tuning. The SVM model achieved an accuracy of 85.02%, which is comparable to the logistic regression model. Like the logistic regression model, the SVM performs well in classifying positive sentiments, with an impressive F1-score of 0.92, and negative sentiments, with an F1-score of 0.78. The model also struggles with neutral sentiments, showing only 33% precision and 2% recall. This indicates the model correctly identifies only 2% of all actual neutral reviews, and one-third of the predictions it makes for neutral reviews are accurate.

**Table 10:** Support Vector Machine Model Performance.

Support Vector Machine			
Metrics	Negative	Neutral	Positive
Support	3261	941	9350
Precision	0.75	0.33	0.89
Recall	0.81	0.02	0.95
F1-Score	0.78	0.03	0.92
Cross-Validation Scores (cv = 5)	0.8441, 0.8512, 0.8504, 0.8516, 0.8539		
Mean Cross-Validation Accuracy	0.8502		

**Table 11:** Support Vector Machine Model Performance After Fine-Tuning.

Support Vector Machine (C = 10, Kernel = ‘Linear’)			
Metrics	Negative	Neutral	Positive
Support	3261	941	9350
Precision	0.74	0.28	0.90
Recall	0.79	0.14	0.92
F1-Score	0.76	0.19	0.91
Cross-Validation Scores (cv = 5)	0.8304, 0.8331, 0.8302, 0.8325, 0.8373		
Mean Cross-Validation Accuracy	0.8327		

To further enhance the performance of the SVM model, we fine-tuned it by adjusting the regularization parameter C and setting the kernel to ‘linear’. Specifically, we increased the regularization parameter (C) to 10 to evaluate its effect on model performance. A higher C value in SVM typically signifies a greater emphasis on minimizing classification errors on the training set, potentially leading to improved model accuracy. Despite these adjustments, the tuned SVM model yielded an accuracy of 83.27%, which is slightly lower than the previous version. However, the larger C model shows a slight improvement in neutral sentiment detection, but overall performance remains low. In summary, while increasing C slightly improves the detection of neutral sentiments, it results in a decrease in overall accuracy, suggesting a potential overfitting to the training data.

### 4.2. Performance analysis on deep learning models

#### 4.2.1. Convolutional neural network.

A CNN was employed to perform sentiment analysis and achieved an accuracy of 85.07%. The CNN model was initially set to train for 20 epochs but stopped early at the 7th epoch due to the Early Stopping criterion. Early Stopping was configured with a patience of 3 epochs, meaning the training would halt if no improvement in validation loss was observed for three consecutive epochs. The relatively small number of trainable parameters in the CNN architecture and the structured nature of the dataset contributed to rapid convergence. By the final epoch, the training accuracy reached 93.13%, while the validation accuracy had peaked earlier at 85.72% during the second epoch, indicating potential overfitting. Although the number of epochs completed was low, this was driven by clear validation trends suggesting that further training would not yield improved generalization.

To enhance the performance, the fine-tuned CNN model included additional layers compared to the initial model. Firstly, a SpatialDropout1D layer with a dropout rate of 0.2 was added after the embedding layer to reduce overfitting. Then, two Conv1D layers were included: the first with 128 filters and the second with 64 filters, both with a kernel size of 5 and Rectified Linear Unit (ReLU) activation. Each Conv1D layer was followed by a MaxPooling1D layer and a Dropout layer with a 0.5 dropout rate to prevent overfitting. Additionally, the fine-tuned model included an extra Dense layer with 256 units and ReLU activation, followed by another Dropout layer, before the final Dense layer with 128 units and the output layer. These modifications aimed to enhance the model’s ability to capture complex patterns in the data and improve generalization.

**Fig 14:**
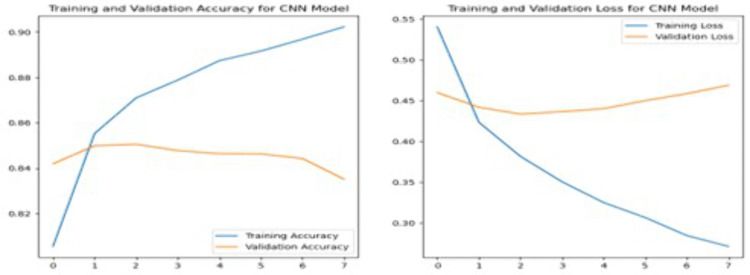
Left: Training and Validation Accuracy for Enhanced Convolutional Neural Network Model, Right: Training and Validation Loss for Enhanced Convolutional Neural Network Model.

Fig 14 suggests that during the initial epochs, training accuracy steadily increases while training loss decreases, demonstrating effective learning. However, starting from Epoch 2, the validation loss begins to rise, and validation accuracy starts to decline, indicating overfitting. This trend suggests that the model becomes increasingly specialized to the training data and loses generalization capability on the validation set. Compared to the previous model, the fine-tuned CNN model achieved a slightly lower accuracy of 84.51%.

#### 4.2.2. Recurrent neural network.

**Fig 15:**
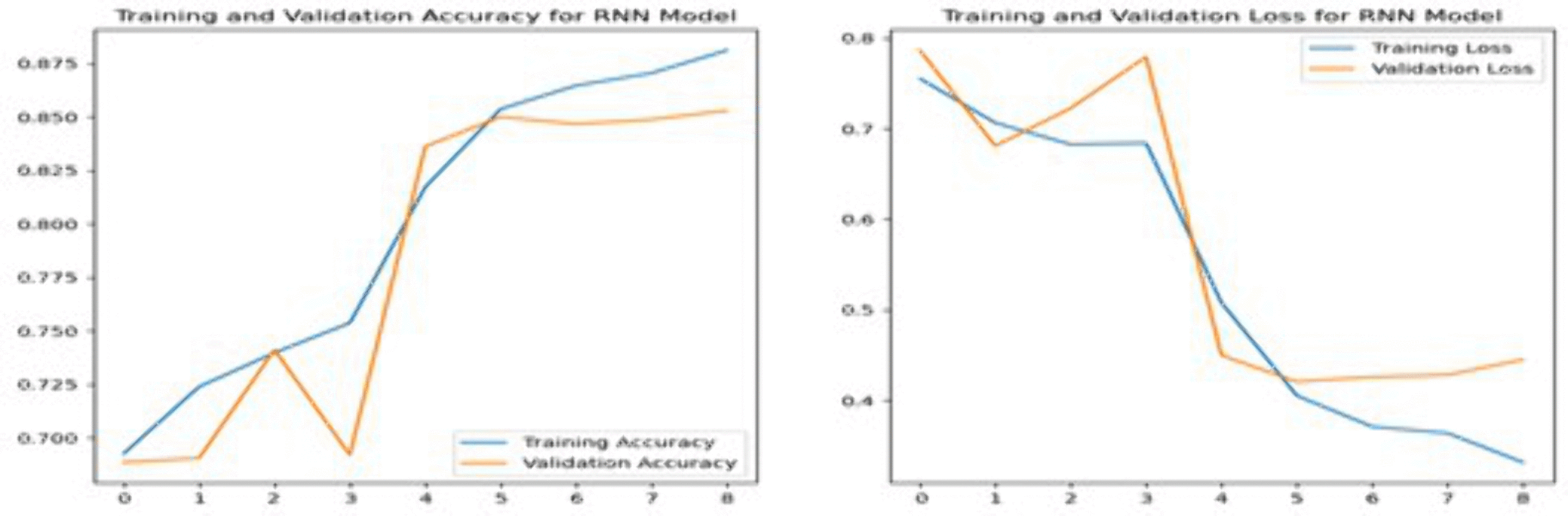
Left: Training and Validation Accuracy for Enhanced Recurrent Neural Network Model, Right: Training and Validation Loss for Enhanced Recurrent Neural Network Model.

When evaluated on the test set, the RNN model achieved an accuracy of 84.56%, which is comparable to the CNN model. While the RNN showed improvement in accuracy over epochs during training, the trends displayed in Fig 15 reveal signs of early instability and eventual overfitting. From Epochs 0–3, both training and validation accuracy fluctuate noticeably, suggesting that the model struggled to generalize consistently during the early stages of training. A significant gain in performance is observed from Epoch 4 onward, culminating in peak validation accuracy around Epochs 6 and 7. However, validation accuracy begins to decline slightly after Epoch 8, even as training accuracy continues to rise, indicating that the model starts to overfit to the training data.

To further improve the model, an enhanced RNN configuration was implemented by introducing an additional LSTM layer with 100 units. This LSTM layer was paired with dropout and recurrent dropout rates of 0.3 to reduce overfitting. The additional layer helped capture more complex sequential dependencies in the input text. This was followed by a Dense layer with 128 units and Rectified Linear Unit (ReLU) activation to extract additional features, and a final Dense layer with SoftMax activation for three-class sentiment classification.

As shown in the training plots, training and validation loss both decline steadily up to Epoch 6. After this point, training loss continues to drop, while validation loss begins to plateau and slightly increase from Epoch 8 onward. Similarly, training accuracy steadily increases across all epochs, but validation accuracy peaks around Epochs 7–8 and shows a slight decline afterward. These trends clearly indicate that while the model initially generalizes well, overfitting begins to emerge in the later epochs. The enhanced RNN model achieved a slightly improved accuracy of 85.01% on the test set, reflecting marginal but consistent gains over the baseline.

The graphs indicate early instability followed by convergence and later-stage overfitting, as shown by the divergence between training and validation metrics after Epoch 8. The performance instability observed in the RNN model, particularly during the early and late training epochs, may be attributed to suboptimal hyperparameter settings or the model’s architectural complexity relative to the dataset. Although dropout techniques were employed to mitigate overfitting, the model’s generalization capacity appeared limited after a certain point in training. Further optimization, such as adjusting learning rates, increasing data volume, or exploring simplified architectures, may enhance performance stability in future work.

**Fig 16:**
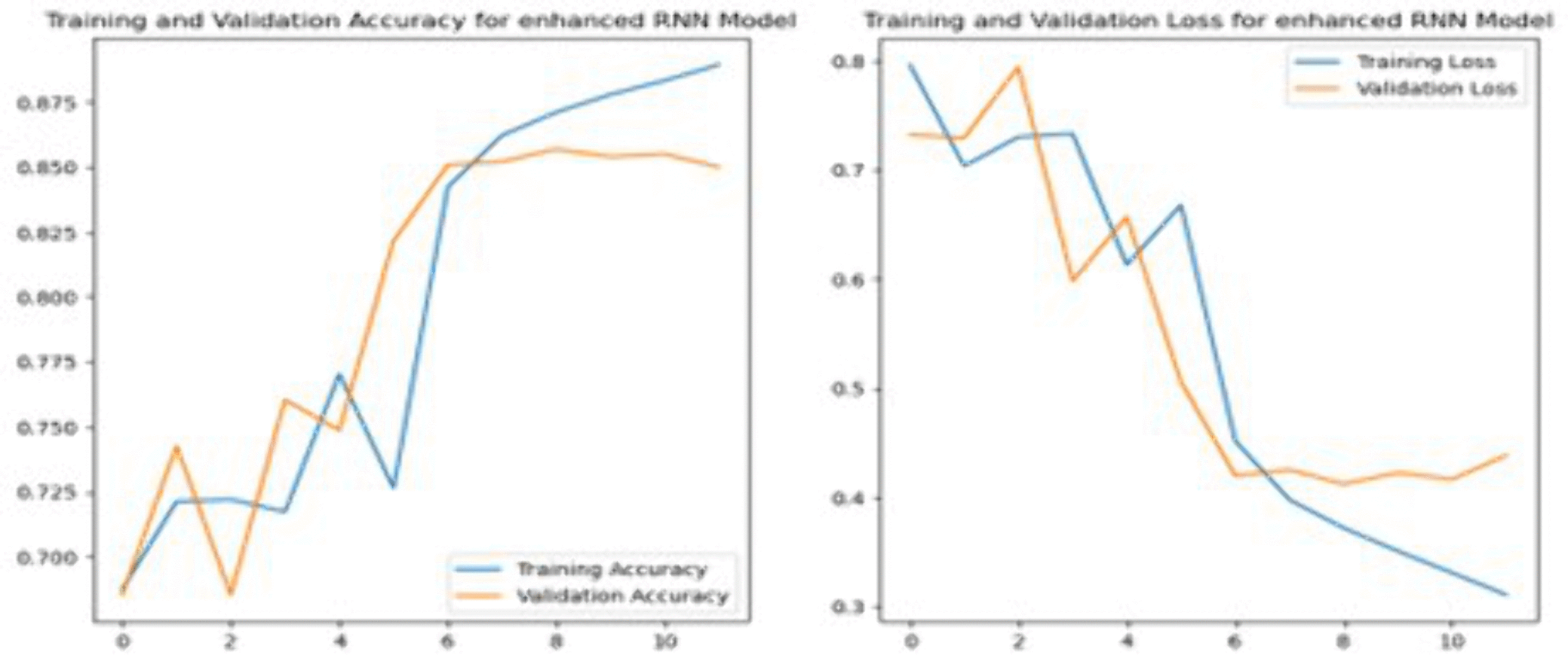
Enhanced Recurrent Neural Network Model Accuracy and Loss in Training.

To further improve the model, an enhanced RNN model was designed by adding an additional LSTM layer with 100 units. This layer is paired with dropout and recurrent dropout rates of 0.3 to help prevent overfitting. The addition of the extra LSTM layer allows the model to further capture more complex patterns and relationships within the data. Following the LSTM layers, the model includes a Dense layer with 128 units and ReLU activation to extract further features, and a final Dense layer with SoftMax activation for three-class sentiment classification. Initially, the model shows steady improvements, with both accuracy and loss trending positively as shown in Fig 16. Nevertheless, from Epochs 1–3, there is some fluctuation in the validation metrics, indicating instability in the learning process. A notable improvement occurs after Epoch 4, where validation accuracy increases significantly. Yet, after Epoch 9, the validation metrics begin to decline slightly, suggesting potential overfitting as the model continues to improve on the training set but shows less progress on the validation set. This indicates that the model’s generalization capacity may be nearing its limit, with the best performance observed around Epoch 8. The model achieved an accuracy of 85.01% on the test set, showing only a slight improvement over the previous RNN model.

#### 4.2.3. Long short-term memory.

**Fig 17:**
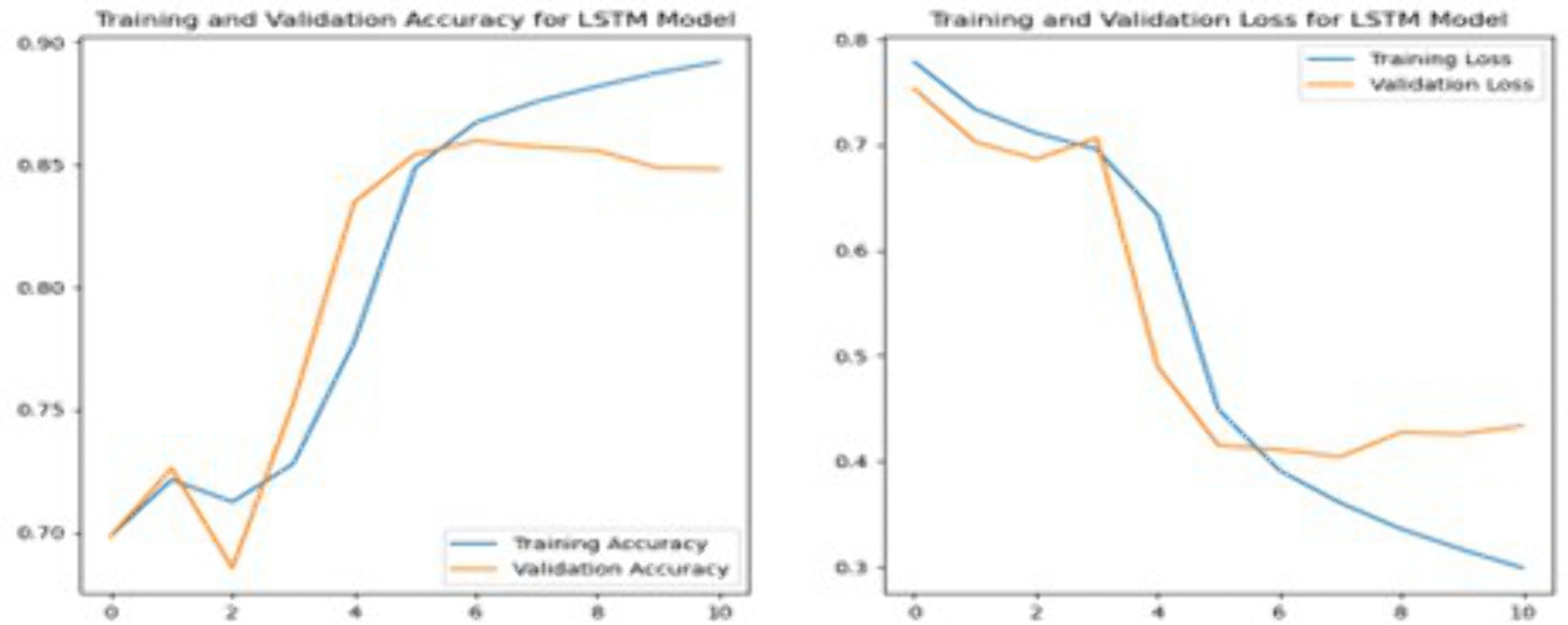
Long Short-Term Memory Model Accuracy and Loss in Training.

The initial LSTM model’s training performance shows an improvement over epochs, though with some fluctuations. According to Fig 17, the model’s accuracy gradually increases as the loss decreases from Epoch 1 to Epoch 4, reflecting a positive learning curve. A significant improvement occurs around Epoch 5, where the model’s accuracy increases, especially in validation accuracy, which increase from 70% to 85%. This trend continues as the model reaches its peak performance in Epoch 7, where the validation accuracy stabilizes around 85.97%. Conversely, in subsequent epochs, the model’s performance starts to drop, with minor increases in accuracy and slight increases in validation loss, indicating potential overfitting. The final accuracy achieved on the test set is 85.04%, which suggests the model is may have limited further improvement beyond this point.

**Fig 18:**
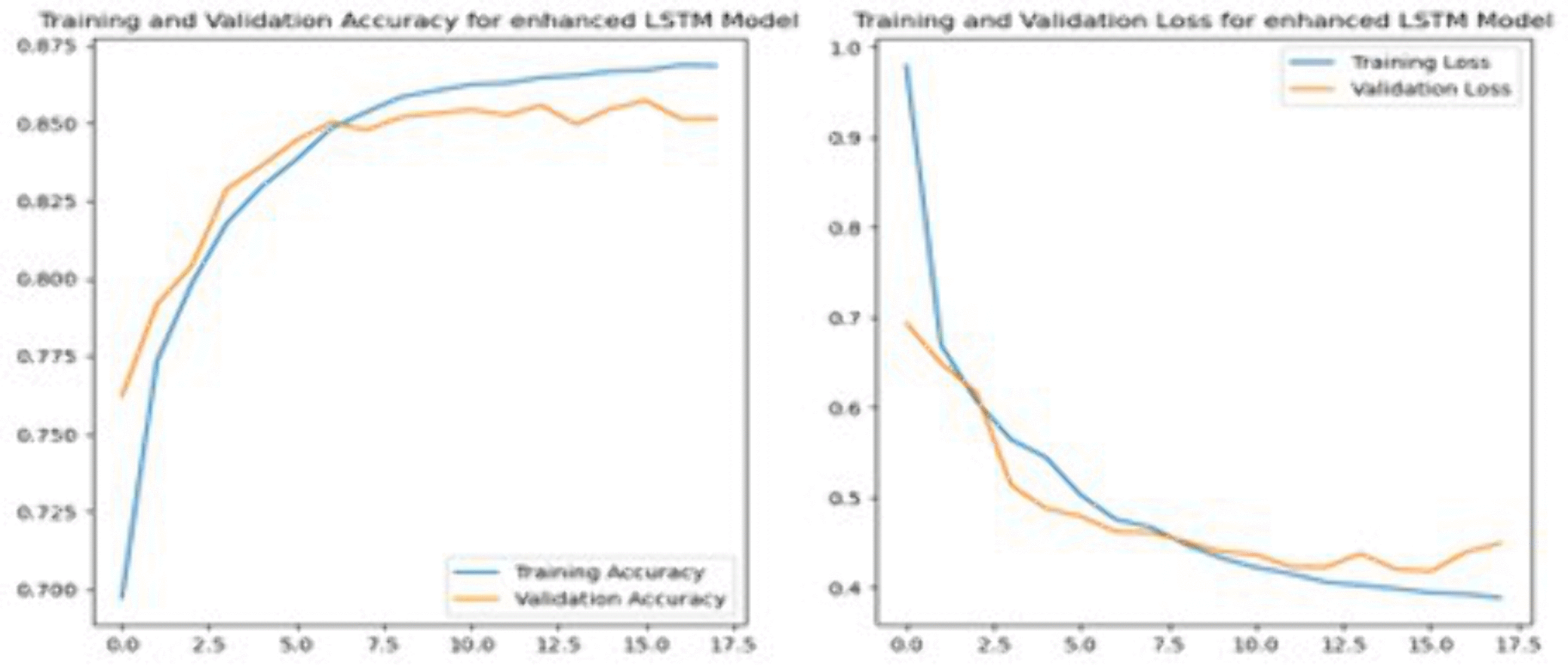
Enhanced Long Short-Term Memory Model Accuracy and Loss in Training.

The enhanced LSTM model uses a slightly reduced learning rate (0.0005), which appears to stabilize the learning process more effectively. Validation accuracy and loss metrics demonstrate a more consistent improvement across epochs as presented in Fig 18. The validation accuracy continues to improve up to Epoch 16 before showing signs of minor fluctuations, while the first model started showing signs of overfitting by Epoch 9. Both models ultimately achieve similar final test accuracies of 85%. Yet, the second model achieves this through a more stable learning process, suggesting potentially better generalization to unseen data, even though the improvement in accuracy is minimal. In summary, the enhanced LSTM model shows improvements in training stability, overfitting control, and consistent performance across epochs. Although both models achieve similar final accuracies, the second model’s training process suggests a better-tuned model with improved learning dynamics.

### 4.3. Classification model performance comparison

To compare the performance of various models on a sentiment classification task, the overall accuracy of each model is provided in Table 12 below. These accuracy scores provide a straightforward measure of how well each model performs in correctly classifying sentiment across a large dataset. Following the accuracy comparison, Table 13 and Table 14 offer a more granular analysis by presenting key performance metrics, including precision, recall, F1 score, and support for each sentiment class (negative, neutral, and positive). These tables provide insight into the strengths and weaknesses of each model, not just in terms of overall accuracy, but in their ability to manage the nuances of sentiment classification across various categories.

**Table 12:** Accuracy Score for The Models.

Model	Accuracy
Decision Tree Model after fine tuning	0.7731
Naive Bayes Model after fine tuning	0.8269
Logistic Regression Model after fine tuning	0.8507
Support Vector Machine	0.8502
Convolutional Neural Network Model	0.8507
Enhanced Recurrent Neural Network Model	0.8490
Enhanced Long Short-Term Memory Model	0.8505

**Table 13:** Classification Report for Traditional Classification Models.

Class	Classification	Precision	Recall	F1 Score	Support
Negative	Decision Trees	0.66	0.66	0.66	3261
	Naïve Bayes	0.78	0.71	0.74	
	Logistic Regression	0.77	0.80	0.78	
	Support Vector Machine	0.75	0.81	0.78	
Neutral	Decision Trees	0.23	0.17	0.20	941
	Naïve Bayes	0.24	0.04	0.07	
	Logistic Regression	0.28	0.03	0.05	
	Support Vector Machine	0.33	0.02	0.03	
Positive	Decision Trees	0.86	0.88	0.87	9350
	Naïve Bayes	0.86	0.96	0.90	
	Logistic Regression	0.89	0.95	0.92	
	Support Vector Machine	0.89	0.95	0.92	

**Table 14:** Classification Report for Deep Learning Models.

Class	Classification	Precision	Recall	F1 Score	Support
Negative	CNN Model	0.74	0.84	0.79	3307
	RNN Model	0.72	0.83	0.77	
	LSTM Model	0.76	0.82	0.79	
Neutral	CNN Model	0.41	0.02	0.04	1005
	RNN Model	0.30	0.01	0.02	
	LSTM Model	0.00	0.00	0.00	
Positive	CNN Model	0.90	0.94	0.92	9240
	RNN Model	0.89	0.94	0.91	
	LSTM Model	0.88	0.96	0.92	

When examining the accuracy scores, the Logistic Regression and CNN models achieved the highest overall accuracy at 85.07%, slightly outperforming LSTM (85.05%), and SVM (85.02%). Although the differences in accuracy are relatively small, these results indicate that even slight variations in model architecture or algorithm choice can impact performance in sentiment analysis tasks.

The comparative performance of traditional classification models, Decision Trees, Naive Bayes, Logistic Regression, and SVM across different sentiment classes highlights significant differences in effectiveness. For negative sentiments, both Logistic Regression and SVM demonstrate impressive performance, each achieving an F1 Score of 0.78. The Decision Trees model, however, shows more modest performance with an F1 Score of 0.66. Regarding neutral sentiments, the Decision Trees model performs best among the models with an F1 Score of 0.20, but this reflects a limitation in capturing neutral sentiments effectively, as it recalls only 17% of these cases. The other models perform even worse, particularly in terms of recall, with values falling below 0.05. This underscores the difficulty all models face in accurately classifying neutral sentiments. In summary, Logistic Regression and SVM are particularly effective for classifying both positive and negative sentiments, while they struggle with neutral sentiments. The Decision Trees model performs slightly better on neutral sentiments compared to others but is still limited. The overall effectiveness of Logistic Regression and SVM makes them more robust for handling sentiment classification, although the underrepresentation of neutral sentiments in the training data likely contributes to the models’ struggles with accurately identifying and classifying these less frequent cases.

The evaluation of DL models including CNN, RNN, and LSTM models reveals performance characteristics across the sentiment categories of negative, neutral, and positive. For negative sentiment classification, all models exhibit good performance, with an average F1 Score of 0.77. For positive sentiment classification, all three models perform strongly. The CNN model achieves an F1 Score of 0.92, with high precision of 0.90 and recall of 0.94. The RNN model follows closely with an F1 Score of 0.91, precision of 0.89, and recall of 0.94. The LSTM model also performs well, reaching an F1 Score of 0.92 with a precision of 0.88 and recall of 0.96. In contrast, classifying neutral sentiments remains challenging for all models. Each model reports extremely low scores across precision, recall, and F1 Score for this category. The CNN model shows a slightly better performance with a precision of 0.41 and recall of 0.02, but these figures remain notably low. The RNN model performs worse with a precision of 0.30 and recall of 0.01, while the LSTM model struggles significantly, with all metrics at zero. This poor performance in identifying neutral sentiments is likely due to the imbalanced representation of neutral sentiments in the dataset, which provides insufficient context for the models to learn and generalize effectively.

Based on the provided results, the CNN and LSTM Model are the best models due to their strong performance across positive and negative sentiments. Logistic Regression has the highest accuracy but is not the best in all sentiment-specific metrics compared to CNN and LSTM. For neutral sentiment classification, all models are faced with challenges. If overall accuracy and balanced performance across sentiment categories are prioritized, the CNN Model might be preferred due to high F1 Scores in both positive and negative sentiments. The LSTM Model is also a strong candidate as it able to capture positive sentiment with high recall.

#### 4.3.1. Statistical significance analysis.

To evaluate whether the observed accuracy differences are statistically meaningful, a paired t-test was applied to the five-fold cross-validation accuracies of the CNN against each baseline model. Table 15 reports the resulting p-values (df = 4). A threshold of 0.05 was used to determine significance. The CNN outperforms Decision Trees and Naïve Bayes with statistically significant margins, while its advantage over Logistic Regression, SVM, RNN, and LSTM is not significant at the 0.05 level.

**Table 15:** Statistical Significance of Accuracy Differences Between Convolutional Neural Network and Baseline Models (Paired t-test Results).

Comparison Model	p-value	Significant (α = 0.05)
Decision Trees	0.003	Yes
Naïve Bayes	0.011	Yes
Logistic Regression	0.238	No
Support Vector Machine	0.267	No
Recurrent Neural Network	0.164	No
Long Short Term Memory	0.291	No

These results support the descriptive metrics in Table 12: although CNN shares similar mean accuracy with Logistic Regression and LSTM, its superiority is statistically confirmed only over the lower-performing traditional models. Consequently, conclusions about model ranking should emphasise practical as well as statistical considerations.

### 4.4. Brand topic detection result

After classification, the identification of main subjects within the Amazon customer review dataset is facilitated through the utilization of two topic detection algorithms: LDA and NMF. The purpose of this is to reveal the main topics people are talking about in the Amazon customer review dataset.

#### 4.4.1. Result from latent dirichlet allocation.

The LDA model was employed to discover five primary topics for each brand within the Amazon customer reviews dataset. These topics are characterised by a distribution of words that summarise the key themes prevalent in the reviews. The main points include:

- **Overall Satisfaction**: General user satisfaction with phone performance and features across brands.
- **Phone Battery**: Battery performance is a common topic, with users discussing both positive and negative experiences. Brands like Nokia and Oneplus saw discussions around battery life, with some users highlighting long battery life as a positive and charging experiences.
- **Camera Quality**: The camera is another frequently mentioned feature, with users of brands like Huawei, Sony, and Xiaomi discussing the quality of pictures and overall camera performance. This is often tied to their satisfaction or disappointment with the device.
- **Connectivity and Network Issues**: Several brands, including Nokia, Samsung, and OnePlus, have topics related to SIM card functionality and network connectivity, with mixed reviews.
- **Operating System and Updates**: Discussions around operating systems, particularly Android updates, were also prominent, with users sharing their experiences and opinions on software performance and updates.
- **Spanish-speaking users Feedback**: For brands like Samsung, Huawei and Apple, some topics captured feedback from Spanish-speaking users who highlights positive experiences with the products.

By detecting these topics across different brands, the LDA model provides valuable insights into the aspects of phone performance that matter most to consumers. The topic explanations for each brand can be found in Table 1–10 of Appendix A in S1 File.

#### 4.4.2. Result from non-negative matrix factorization.

After that, the NMF model was also employed to discover five primary topics for each brand within the Amazon customer reviews dataset. The key points identified by NMF model include:

- **User Satisfaction**: Most of the users expressed positive experiences with their phones across brands. Many reviews highlighted the value for money, with users satisfied with the product’s quality relative to its price.
- **Camera Performance**: The camera was frequently mentioned, with users noting either satisfaction or concerns about camera quality. This theme was particularly prominent in reviews for brands like Motorola, Huawei, and Xiaomi.
- **Phone Battery**: Phone battery life and charging experiences were common discussion points, with users commenting on the longevity and reliability of their phones’ batteries.
- **Connectivity and Network Issues:** SIM card and network compatibility issues were mentioned, especially in relation to specific carriers like Verizon and T-Mobile. This was a recurring theme across several brands.
- **Gift-Giving**: Many users expressed that they bought phones as gifts for family members, reflecting emotional satisfaction and the perception of these phones as suitable and reliable gifts.
- **Product Condition**: The physical condition of the phones upon arrival, including aspects like scratches was another theme. Users often commented on the phone’s appearance and whether it met their expectations.
- **Mixed Reviews**: Some brands, like Asus, had a mix of positive and negative reviews, particularly around specific models like the Zenfone series, which had varied feedback on design and sound quality.
- **Spanish-speaking users Feedback**: For brands like Huawei and Xiaomi, some topics captured feedback from Spanish-speaking users who highlights positive experiences with the products.

This explanation highlights that the NMF model captured not only performance-related aspects but also emotional themes. The topic explanations for each brand can be found in Tables 11–20 of Appendix B in S1 File.

#### 4.4.3. Performance comparison.

To provide an objective assessment of topic quality, we calculated the CS using the widely adopted c_v metric. Higher CS values indicate greater semantic similarity among the top words in a topic, resulting in more interpretable themes. The LDA model achieved a CS of 0.41, whereas the NMF model obtained a higher CS of 0.54. These quantitative results corroborate the qualitative findings in Sections 4.4.1 and 4.4.2, confirming that the NMF topics are more coherent and therefore better suited for analysing brand perception in this dataset.

In evaluating the performance of these two topic-detection approaches, the NMF model emerges as the most effective for this study. NMF produces more focused, detailed, and interpretable topics, making it the strongest tool for understanding brand perceptions within customer reviews.

The LDA model tends to capture topics that are closely related to phone features yet often overlapping. Themes such as “battery” and “camera” frequently intersect with broader topics like overall user satisfaction, limiting LDA’s ability to separate distinct aspects of user experience, especially in the smartphone domain, where multiple features influence perception simultaneously.

By contrast, the NMF model delivers more precise and distinguishable topics. It not only identifies general themes like user satisfaction but also uncovers specific issues such as product condition upon arrival or mixed feedback on certain models (e.g., Asus Zenfone). NMF further highlights detailed topics such as camera performance and connectivity issues, each treated as a distinct subject with its own sentiment profile. This granularity enables deeper analysis of how customers perceive individual phone features. When NMF reveals, for instance, that camera performance is a significant concern for a brand, product development and marketing teams can address that issue directly. The clarity and specificity of NMF topics thus provide actionable insights, facilitating improved brand management and customer satisfaction.

In conclusion, NMF proves to be the most effective topic-modelling technique for this study. Its capacity to extract distinct, focused topics and capture a wide range of user sentiments and product attributes makes it the preferred choice for understanding and analysing brand perception in customer reviews. By leveraging NMF, business owners gain a deeper and more actionable understanding of how customers perceive different aspects of their products, enabling more targeted improvements and more effective communication with consumers.

### 4.5. Analysis on brand sentiments

After selecting the best topic detection model, brand sentiments were generated by applying the VADER sentiment analysis on the results. Sentiment scores help categorize customer opinions into positive, neutral, or negative, and provide a clear measure of consumer sentiment. The sentiment scores for each brand can be found in Table 21 of Appendix C in S1 File. Here is a summary of the sentiment scores for various smartphone brands:

- **Motorola**: The sentiment for Motorola is mostly positive. The positive sentiment in the topics related to product value and overall phone experience highlights strengths in these areas. However, topics like battery life and network issues indicate areas where improvement could be made. Motorola should focus on addressing these neutral aspects to improve overall sentiment.
- **Nokia:** Nokia’s feedback is predominantly neutral. This suggests a balanced view of the brand and stable customer perception. Positive sentiments are present in discussion about product quality and performance. Nokia should aim to boost its positive sentiment by improving neutral sentiments and enhancing customer experience.
- **Samsung**: Samsung’s sentiment analysis shows a balanced mix of neutral and positive feedback. It is reported that customers are happy with the performance of the phone and the gift selection. However, neutral sentiments suggest that Samsung did not exceed customer expectations. Samsung should work on converting neutral perceptions into positive ones by addressing potential issues related to battery life.
- **HUAWEI**: HUAWEI receives mostly positive sentiment across topics. Customer frequently express strong satisfaction of the phone’s performance, camera quality and overall value. HUAWEI should maintain its focus on these features and customer experience.
- **Sony**: Sony’s sentiment analysis is large positive. The strong positive feedback on features and performance suggests that Sony is performing well in these areas. Sony should continue to enhance and innovate in these strengths.
- **Apple**: Apple’s feedback is mainly neutral. Based on the neutral sentiments, customers are generally satisfied with the phone but were not enthusiastic about it. In general, the impression remains stable, but a low score is given to the issue with the screen. Apple should focus on enhancing features and addressing any minor issues to shift more feedback into the positive category and boost overall customer satisfaction.
- **Google**: Google has mixed sentiments with both neutral and positive feedback. Product performance and value are rated positively, but other areas like screen or charging remain neutral. Google should improve these neutral areas to elevate overall customer perception.
- **ASUS**: ASUS receives positive feedback, with high scores in product quality and performance. Customers are strongly pleased with the phone’s performance and features. ASUS should continue to focus on maintaining the qualities that contribute to this positive feedback and look for opportunities to further enhance the customer experience.
- **OnePlus**: OnePlus exhibits a mostly positive sentiment with positive feedback on product value and performance. The presence of some neutral feedback on screen, battery and camera indicates that there is still room for improvement. OnePlus should address these neutral aspects to further strengthen its positive reputation and customer satisfaction.
- **Xiaomi:** Xiaomi’s sentiment analysis reveals a strong positive reception overall, with high scores in product quality and performance. Despite some neutral feedback, the positive sentiment is dominant. Xiaomi should continue to focus on the strengths that contribute to this positive view while addressing any areas of neutral feedback to enhance overall customer satisfaction.

Overall, these sentiment analyses provide a comprehensive view of customer perceptions, enabling brands to address concerns. This information also can guide strategic decisions, such as enhancing product features that are well-received, addressing common complaints, or refining marketing messages to align with customer expectations.

### 4.6. Visualisation

Following the sentiment analysis and topic detection, which offered a deeper understanding of the underlying themes and sentiments within the dataset, we now shift our focus to visualizing the broader aspects of the data. Visualization is essential for uncovering patterns, trends, and insights. This section presents a series of visualizations that offer a comprehensive overview of the dataset, employing both Python and Tableau for this purpose.

**Fig 19:**
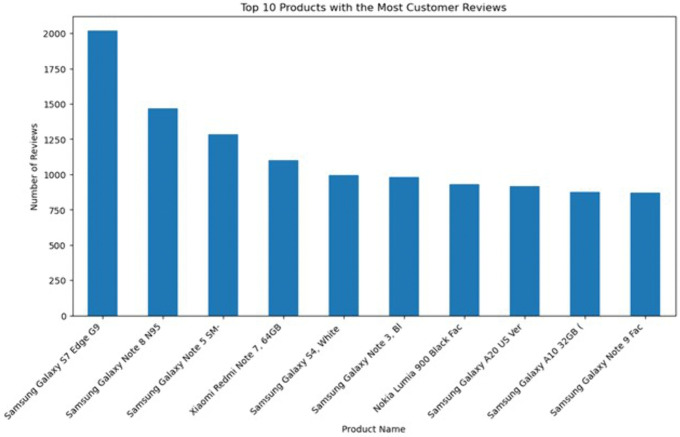
Top 10 Products with Most Customer Reviews.

**Fig 20:**
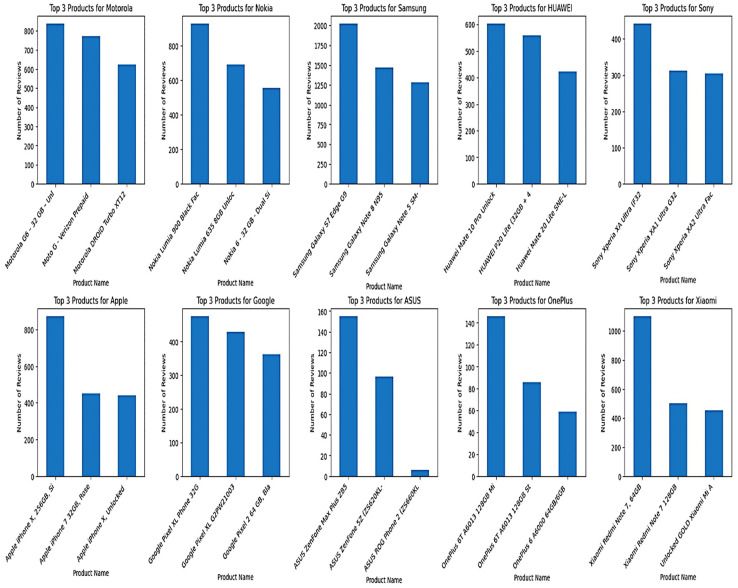
Top 3 Products for Each Brand.

Several graphs were created using Python to provide a detailed analysis of the dataset. These Python-generated graphs offer an initial exploration of the data. The top ten products with the most customer reviews are presented in Fig 19, allowing business owners to identify which products were customer favourites during this timeframe. Most of these products are from the Samsung brand, with only one product each from Xiaomi and Nokia. By knowing the popular brand among the smartphone users, a business owner can determine if a particular feature or product brand resonates more with consumers, enabling them to focus on those aspects in future inventory management and marketing strategies. Besides, Fig 20 plots the top three products for each brand, providing insights into the best-selling products for each brand. This information can help business owners understand which products are most popular among customers for each specific brand. For instance, a business owner can manage product inventory more effectively by deciding whether to stock up on popular items, reduce stock for less favoured ones, or import similar products with popular features.

**Fig 21:**
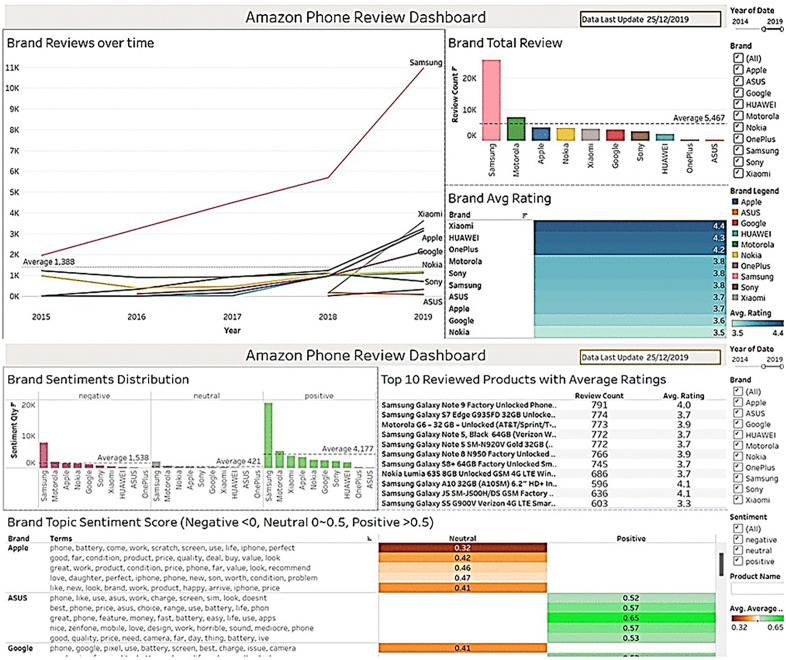
Tableau Dashboard for Amazon Customer Reviews on Phones.

Additionally, a comprehensive dashboard was created in Tableau to compile and extend these insights as seen in Fig 21. The Tableau dashboard includes visualizations such as reviews over time, average ratings by brand, total reviews by brand, sentiment distribution across categories for each brand, and a summary of the top ten products by brand in terms of review count and average rating. This dashboard serves as a powerful tool for gaining a comprehensive view of Amazon customer reviews across distinct brands, allowing for a deeper understanding of brand performance and customer perception.

## 5. Conclusion, limitations, and recommendations

In conclusion, this study addressed the challenge of extracting actionable insights from the extensive volume of customer reviews, particularly within the smartphone industry. By applying sentiment analysis techniques, topic detection, and an interactive Tableau dashboard, this research filled a gap in the literature on brand sentiment analysis for smartphone brands. The results showed that the CNN model, which achieved the highest accuracy at 85.07%, provided balanced performance across sentiment categories and proved to be particularly effective for overall sentiment analysis. Although Logistic Regression achieved similar accuracy, it fell short in comparison to CNN across sentiment-specific metrics. All models struggled with classifying neutral sentiments, which may be attributed to the less emotionally charged nature of these reviews.

While early stopping and dropout were applied to address overfitting in the CNN model, we acknowledge that the observed gap between training and validation accuracy suggests room for further improvement. Due to the scope of the current study, additional regularization techniques such as L2 weight decay, batch normalization, and text augmentation were not explored. These strategies are being investigated in ongoing follow-up work aimed at enhancing model generalization and robustness. All DL models were capped at 20 global epochs to maintain parity of training time across the benchmark. While early stopping indicated convergence in most cases, this cap may have curtailed the models’ ability to extract deeper feature hierarchies. Ongoing work is experimenting with extended epoch schedules, cosine learning-rate decay, and warm-up strategies to determine whether additional training yields measurable gains in generalisation.

Building on these findings, the research made significant contributions. Firstly, the development of an interactive Tableau dashboard enables businesses to monitor customer sentiment trends and brand perception effectively. This tool provides businesses with a comprehensive visual analysis of customer feedback, helping them to make data-driven decisions that enhance brand image and customer satisfaction. Secondly, this study proposed a new framework that applies sentiment analysis, topic detection and brand topic sentiments. This approach provides a holistic view of customer feedback, which is essential for businesses in the competitive smartphone market.

However, certain limitations must be acknowledged. The dataset used only includes information up to 2019, which may not reflect the most recent trends or shifts in language usage. Additionally, challenges in text preprocessing, such as handling multilingual content and slang, may have impacted model performance. Future research should consider using real-time social media analytics, if possible, to capture more up-to-date customer feedback. The dataset also revealed a clear imbalance in sentiment labels. This imbalance posed a risk of model bias during classification. To mitigate this, class weighting was applied during model training, ensuring that minority classes contributed more significantly to the loss function. Preliminary experiments showed that weighted training improved the recall of Negative and Neutral classes by three to five percentage points compared with an unweighted baseline. Techniques such as Synthetic Minority Over-sampling Technique (SMOTE), data augmentation, or ensemble re-weighting were not employed in this study; future work could explore these alternatives to determine whether they further mitigate imbalance without introducing noise.

In this study, we adopted a conventional 80/20 random train-test split to benchmark model performance, which allowed for a balanced distribution across sentiment categories. However, we recognise that this approach may not fully capture the complexities of real-world generalisation. The observed similarity in model performance could, in part, stem from the homogeneity introduced by this split strategy. As such, we acknowledge the value of more challenging evaluation frameworks. Future work will explore brand-wise, temporally stratified, and cross-platform data splits to better evaluate model robustness and generalisability across diverse product lines and evolving consumer contexts. Other sources of imbalance, such as the uneven temporal and brand distribution of reviews, were also not explicitly addressed. For example, some brands only appear in later years (e.g., Xiaomi), which may influence the model’s ability to generalize across brands and time periods. Future work could explore temporal resampling or brand-stratified evaluation to better account for these biases.

Reviews were categorized using an automated Python-based heuristic labelling strategy, which relied in part on the VADER lexicon-based approach. While this facilitated efficient labelling of over 60,000 reviews, it may oversimplify sentiment classification, particularly for neutral or ambiguous cases. Lexicon-driven methods such as VADER are constrained by their dependence on pre-defined word lists and heuristics, which can struggle to capture contextual nuances, sarcasm, or domain-specific terminology. These limitations introduce potential ambiguity in the dataset and may affect model training and evaluation outcomes. Future iterations of this study may therefore explore more robust labelling methods, such as manually annotated datasets, semi-supervised learning approaches, or transformer-based sentiment labelling techniques, to improve contextual sensitivity and reliability.

Due to computational constraints, hyperparameter tuning for CNN, RNN, and LSTM models was limited to a focused grid of empirically selected values. While this ensured fair benchmarking, we acknowledge that a broader search might have yielded better-optimised configurations. Each model was tuned via five-fold cross-validation, with the best settings selected based on mean validation accuracy. In future work, we aim to adopt more systematic tuning methods, such as Bayesian or random search, to explore a wider range of architectural and optimisation parameters and further improve model robustness. While the current pipeline relies on conventional ML and foundational DL architectures, recent advances in NLP demonstrate that richer text representations can be obtained through Word Embeddings such as Word2Vec and GloVe, and through Transformer-based LLM such as BERT, Robustly Optimized BERT Pretraining Approach (RoBERTa), and GPT. These models capture context-dependent semantics, idiomatic expressions, and subtle sentiment cues that conventional bag-of-words and static embeddings may overlook. Future work will therefore integrate pre-trained contextual embeddings or fine-tuned transformer encoders into the sentiment-classification stage, benchmark their performance against the current CNN baseline, and evaluate the trade-offs among accuracy, interpretability, and computational cost.

While the present work focuses on Amazon smartphone reviews, we recognize that broader applicability requires careful consideration. The proposed pipeline is adaptable to other e-commerce platforms and consumer domains where reviews share structural similarities, such as hospitality, consumer electronics, and healthcare. However, differences in linguistic styles, platform-specific rating systems, and domain-specific terminology may limit direct transferability. To strengthen generalizability, future research should evaluate the framework across multiple datasets and platforms, potentially using domain adaptation or transfer learning techniques to account for variations in language and context. This would ensure that the approach remains robust and reliable when applied to diverse industries beyond the Amazon ecosystem.

Beyond technical performance, this study also acknowledges the ethical implications of deploying automated sentiment analysis systems. Bias in training data, such as imbalanced sentiment classes, skewed brand representation, or culturally specific language, can propagate through the model and result in unfair or inaccurate predictions. For instance, reviews written in non-standard dialects or by speakers from diverse linguistic backgrounds may be misclassified due to underrepresentation in the training set. Automated sentiment classifiers may also inadvertently amplify stereotypes or marginalize minority voices if not properly audited. To ensure responsible AI practices, future work should incorporate fairness evaluation metrics, conduct bias audits, and explore mitigation techniques such as adversarial debiasing or fairness-aware training. Transparency, accountability, and inclusivity should be core principles in the development and deployment of sentiment analysis models used for real-world decision-making.

In conclusion, this study contributes to a deeper understanding of customer sentiment analysis and provides practical tools for businesses to improve their brand perception and customer satisfaction in the highly competitive smartphone market. At the same time, the ethical implications and challenges of generalizability must not be overlooked. By embedding fairness, transparency, and adaptability into future work, sentiment analysis frameworks can achieve both technical robustness and responsible application across diverse domains.

## Supporting information

S1_File Contains 21 supplementary tables organised under three appendices. **Appendix A:** Topic explanations for each brand using the LDA model. **Table I: LDA Topic Detection for Motorola Brand. Table II: LDA Topic Detection for Nokia Brand. Table III: LDA Topic Detection for Samsung Brand. Table IV: LDA Topic Detection for Huawei Brand. Table V: LDA Topic Detection for Sony Brand. Table VI: LDA Topic Detection for Apple Brand. Table VII: LDA Topic Detection for Google Brand. Table VIII: LDA Topic Detection for Asus Brand. Table IX: LDA Topic Detection for OnePlus Brand. Table X: LDA Topic Detection for Xiaomi Brand**. **Appendix B:** Topic explanations for each brand using the NMF model. **Table XI: NMF Topic Detection for Motorola Brand. Table XII: NMF Topic Detection for Nokia Brand. Table XIII: NMF Topic Detection for Samsung Brand. Table XIV: NMF Topic Detection for Huawei Brand. Table XV: NMF Topic Detection for Sony Brand. Table XVI: MF Topic Detection for Apple Brand. Table XVII: NMF Topic Detection for Google Brand. Table XVIII: NMF Topic Detection for Asus Brand. Table XIX: NMF Topic Detection for OnePlus Brand. Table XX: NMF Topic Detection for Xiaomi Brand**. **Appendix C:** Topic-based sentiment scores for each brand using the VADER method. **Table XXI: Topic-Based Sentiment Score for each brand using VADER.**
(DOCX)
