# Supplementary material for: Decoding brand sentiments: Leveraging customer reviews for insightful brand perception analysis using natural language processing and Tableau
Source: PLoS One. 2025 Dec 4;20(12):e0334330. doi: 10.1371/journal.pone.0334330 (PMC12677512; doi:10.1371/journal.pone.0334330)
Supplement: S1_File — Appendix A: Topic explanations for each brand using the LDA model. Table I: LDA Topic Detection for Motorola Brand. Table II: LDA Topic Detection for Nokia Brand. Table III: LDA Topic Detection for Samsung Brand. Table IV: LDA Topic Detection for Huawei Brand. Table V: LDA Topic Detection for Sony Brand. Table VI: LDA Topic Detection for Apple Brand. Table VII: LDA Topic Detection for Google Brand. Table VIII: LDA Topic Detection for Asus Brand. Table IX: LDA Topic Detection for OnePlus Brand. Table X: LDA Topic Detection for Xiaomi Brand. Appendix B: Topic explanations for each brand using the NMF model. Table XI: NMF Topic Detection for Motorola Brand. Table XII: NMF Topic Detection for Nokia Brand. Table XIII: NMF Topic Detection for Samsung Brand. Table XIV: NMF Topic Detection for Huawei Brand. Table XV: NMF Topic Detection for Sony Brand. Table XVI: MF Topic Detection for Apple Brand. Table XVII: NMF Topic Detection for Google Brand. Table XVIII: NMF Topic Detection for Asus Brand. Table XIX: NMF Topic Detection for OnePlus Brand. Table XX: NMF Topic Detection for Xiaomi Brand. Appendix C: Topic-based sentiment scores for each brand using the VADER method. Table XXI: Topic-Based Sentiment Score for each brand using VADER. (DOCX) [file pone.0334330.s001.docx]

## **Appendices**

## Appendix A Topic Explanations for Each Brand Using the LDA Model

####

#### **Table I: LDA Topic Detection for Motorola Brand**

| **Topic for motorola** |  |  |
| --- | --- | --- |
| **Topic No** | **Keywords** | **Explanation** |
| 1 | Phone, great, love, work, get, moto, good, use | This topic discusses positive reviews related to general phone experiences, highlighting users' satisfaction with the Motorola phone. |
| 2 | Phone, battery, great, good, use, life, camera, like | This topic focuses on the phone's camera and battery performance. |
| 3 | Phone, work, get, one, motorola, new, use, would | This topic reflects user experiences with new Motorola phones, including their performance and functionality. |
| 4 | Phone, use, android, moto, get, update, time, one | This topic likely discusses the Android operating system updates on Motorola phones, including users' experiences and opinions on these updates. |
| 5 | Phone, work, verizon, sim, sprint, card, network, unlocked | This topic addresses phone connectivity issues related to SIM cards, network providers (like Verizon and Sprint). |

#### **Table II: LDA Topic Detection for Nokia Brand**

| **Topic for Nokia** |  |  |
| --- | --- | --- |
| **Topic No** | **Keywords** | **Explanation** |
| 1 | Phone, la, de, charge, work, el, Nokia, port, one | This topic discusses phone charging and overall functionality. |
| 2 | Phone, use, apps, camera, like, Nokia, well, get, window | This topic appears to focus on the performance of apps and the camera on Nokia phones. |
| 3 | Phone, android, great, good, Nokia, price, battery, get, quality | This topic reflects the value of Nokia phones, particularly emphasising good price and battery quality. |
| 4 | Phone, nokia, work, get, use, great, one, charge, time | This topic likely discusses Nokia phone charging experiences. |
| 5 | Phone, sim, work, card, good, Nokia, use, tmobile, call | This topic addresses phone connectivity issues related to SIM cards, network providers (like T-Mobile), and call quality. |

#### **Table III: LDA Topic Detection for Samsung Brand**

| **Topic for Samsung** |  |  |
| --- | --- | --- |
| **Topic No** | **Keywords** | **Explanation** |
| 1 | Phone, great, love, good, work, price, new, like, far, well | This topic highlights positive reviews about new Samsung phone experiences. |
| 2 | Phone, work, get, sim, new, verizon, card, samsung, use, one | This topic addresses phone connectivity issues related to SIM cards and network providers like Verizon. |
| 3 | phone, battery, use, get, call, charge, time, day, work, one | This topic reflects on the phone's battery performance. |
| 4 | Phone, screen, use, note, samsung, camera, well, like, get, battery | This topic discusses various phone features, including the screen, camera, and battery performance. |
| 5 | El, de, muy, la, que, en, lo, excelente  Telefono, con | This topic likely covers general phone experiences from Spanish-speaking users, with an emphasis on overall satisfaction. |

#### **Table IV: LDA Topic Detection for Huawei Brand**

| **Topic for Hua Wei** |  |  |
| --- | --- | --- |
| **Topic No** | **Keywords** | **Explanation** |
| 1 | Phone, great, love, camera, good, price, screen, best, fast, battery | This topic highlights positive reviews about the overall phone experience, focusing on aspects like camera, price, screen, and battery performance. |
| 2 | De, el, la, que, muy, en, lo, excelente, telefono, con | This topic reflects the positive phone experience from Spanish-speaking users. |
| 3 | Phone, use, good, work, get, wifi, Huawei, like, great, call | This topic discusses phone connectivity issues related to Wi-Fi and call quality. |
| 4 | Phone, Huawei, get, use, mate, like, battery, well, work, camera | This topic is focused on specific Huawei models, like the Huawei Mate, discussing aspects like battery life and camera performance. |
| 5 | Phone, camera, good, use, screen, great, work, battery, well, come | This topic covers general phone experiences like Topic 1. |

#### **Table V: LDA Topic Detection for Sony Brand**

| **Topic for Sony** |  |  |
| --- | --- | --- |
| **Topic No** | **Keywords** | **Explanation** |
| 1 | Phone, sony, screen, work, great, get, month, xperia, problem, warranty | This topic discusses issues related to the phone screen, including problems and warranty concerns. |
| 2 | Phone, use, sony, get, one, like, work, camera, dont, would | This topic reflects general user experiences with the phone, including aspects like usability and camera performance. |
| 3 | Phone, screen, use, sony, camera, card, battery, get, time, xperia | This topic covers phone features such as screen quality, camera performance, and battery life. |
| 4 | Phone, camera, use, screen, sony , battery, well, xz, get, charge | This topic is similar to Topic 3, focusing on specific features of the phone. |
| 5 | Phone, great, good, sony, camera  , use, love, like, battery, get | This topic is also like Topic 3. |

#### **Table VI:** **LDA Topic Detection for Apple Brand**

| **Topic for Apple** |  |  |
| --- | --- | --- |
| **Topic No** | **Keywords** | **Explanation** |
| 1 | El, la, de, en, que, lo, muy, esta, un, bateria | This topic discusses phone experiences from Spanish-speaking users. |
| 2 | Phone, battery, good, get, come, use, work, life, condition, review | This topic focuses on the condition and performance of the phone's battery, including user reviews and overall satisfaction. |
| 3 | Phone, work, get, unocked, screen, come, sim, would, one, iphone | This topic reflects on features related to the phone's screen and SIM card, including issues with unlocking and general functionality |
| 4 | Phone, new, like, come, sratch, work, would, brand, screen, buy | This topic discusses negative experiences related to screen scratches on new phones. |
| 5 | Phone, work, great, iphone, new, love  Like, condition, battery, look | This topic is similar to the topic 2. |

####

#### **Table VII:** **LDA Topic Detection for Google Brand**

| **Topic for Google** |  |  |
| --- | --- | --- |
| **Topic No** | **Keywords** | **Explanation** |
| 1 | Phone, google, work, get, charger, one, pixel, use, charge, cable | This topic discusses aspects related to the Google Pixel series, focusing on the functionality and issues with the charger and cable. |
| 2 | Phone, work, new, great, come, get, good, use, like, pixel | This topic highlights the condition and performance of new Google Pixel phones. |
| 3 | Phone, google, issue, get, screen, pixel, work, great, use, day | This topic reflects on screen issues with Google Pixel phones. |
| 4 | Phone, battery, pixel, use, get, work, one, life, day, google | This topic is likely to discuss battery performance and life of the Google Pixel phones, including user feedback on battery longevity and efficiency. |
| 5 | Phone, pixel, google, like, camera, use, battery, android, well, screen | This topic focuses on positive experiences with the Google Pixel phones, emphasising features such as the camera, screen, and battery performance. |

####

#### **Table VIII:** **LDA Topic Detection for Asus Brand**

| **Topic for Asus** |  |  |
| --- | --- | --- |
| **Topic No** | **Keywords** | **Explanation** |
| 1 | Phone, would, asus, like, work, device, zenfone, great, one, update | This topic discusses the Asus Zenfone series. |
| 2 | Phone, call, use, battery, wifi, screen, great, sim, good, charge | This topic focuses on various features of the phone such as battery life, screen quality, SIM functionality, and Wi-Fi performance. |
| 3 | Phone, it, even, asus, camera, look, Like, also, sound, would | This topic reflects on issues or user experiences related to the phone’s camera, screen, and sound quality after use. |
| 4 | Phone, get, use, camera, one, good, great, call, well, work | This topic is likely to discuss the camera performance of Asus phones. |
| 5 | Phone, great, get, use, price, asus, battery, work, best, good | This topic highlights positive aspects of Asus phones. |

#### **Table IX:** **LDA Topic Detection for OnePlus Brand**

| **Topic for OnePlus** |  |  |
| --- | --- | --- |
| **Topic No** | **Keywords** | **Explanation** |
| 1 | Phone, oneplus, camera, get, use, say, great, fast, well, work | This topic discusses user satisfaction with the OnePlus phone camera, including its performance and speed. |
| 2 | Phone, sim, like, oneplus, work, use  , card, new, well, come | This topic focuses on the phone’s SIM card functionality and connectivity. |
| 3 | Phone, great, use, work, good, screen, get, charge, love, battery | This topic reflects on the phone’s battery performance and screen quality. |
| 4 | Phone, oneplus, even, version, like, screen, one, look, op, battery | This topic is likely to discuss aspects related to the phone’s screen and battery, as well as user experiences with different versions of OnePlus phones. |
| 5 | Phone, get, one, screen, use, work, fast, best, charger, android | This topic highlights the phone’s screen quality, charging conditions, and overall performance, particularly Android operating system. |

#### **Table X:** **LDA Topic Detection for Xiaomi Brand**

| **Topic for XiaoMi** |  |  |
| --- | --- | --- |
| **Topic No** | **Keywords** | **Explanation** |
| 1 | Good, phone, product, charger, nice, price, quality, work, really, great | This topic discusses user satisfaction with the phone’s performance, quality, and value for money, including the charger. |
| 2 | phone, camera, well, use, screen, like, good, get, work, one | This topic expresses user experiences with the phone's camera and screen quality, as well as its overall performance. |
| 3 | Phone, great, good, camera, battery, work, get, price, use, love | This topic is similar to Topic 1 but with an emphasis on camera and battery. |
| 4 | Phone, android, use, work, product, get, well, one, ad, fast | This topic discusses the experience with the Android operating system on the phone, including its performance and how it integrates with the device. |
| 5 | De, el, la, que, excelente, muy, en, lo, con, precious | This topic likely covers user satisfaction with the phone from Spanish-speaking users. |

## Appendix B Topic Explanations for Each Brand Using the NMF Model

#### **Table XI:** **NMF Topic Detection for Motorola Brand**

| **Topic for Motorola** |  |  |
| --- | --- | --- |
| **Topic No** | **Keywords** | **Explanation** |
| 1 | phone, battery, use, like, screen, charge, moto, life, day, new | This topic discusses user experiences with the new phone's battery life, screen quality, and overall daily usage. |
| 2 | good, product, price, far, phone, quality, value, really, camera, condition | This topic highlights positive reviews focusing on the camera performance. |
| 3 | great, price, phone, product, value, deal, fast, camera, life, condition | This topic is similar to Topic 2. |
| 4 | love, phone, son, absolutely, daughter, awesome, bought, perfect, moto, husband | The topic expresses users' satisfaction with the phone as a perfect gift for family members. |
| 5 | work, verizon, perfectly, fine, perfect, sim, network, didnt, stop, doesnt | This topic discusses the phone's connectivity, specifically mentioning compatibility with Verizon and issues with SIM cards and network performance. |

#### **Table XII:** **NMF Topic Detection for Nokia Brand**

| **Topic for Nokia** |  |  |
| --- | --- | --- |
| **Topic No** | **Keywords** | **Explanation** |
| 1 | phone, use, nokia, like, work, window, apps, screen, battery, camera | This topic discusses user experiences with Nokia phones, focusing on features like Windows OS, apps, screen quality, battery life, and camera performance. |
| 2 | good, price, product, phone, quality, work, really, far, buy, look | This topic highlights positive reviews about the product's price and quality. |
| 3 | great, phone, price, work, product, value, fast, buy, money, awesome | The subject of this topic emphasises the phone's excellent value for money like the one in Topic 2. |
| 4 | love, phone, window, wife, new, son, bought, perfect, husband, amaze | This topic expresses users' love for Nokia phones as perfect gifts for family members |
| 5 | excellent, product, seller, price, thanks, condition, thank, item, venezuela, delivery | This topic discusses positive feedback on the product's condition and delivery. |

#### **Table XIII:** **NMF Topic Detection for Samsung Brand**

| **Topic for Samsung** |  |  |
| --- | --- | --- |
| **Topic No** | **Keywords** | **Explanation** |
| 1 | phone, like, use, new, battery, screen, samsung, nice, come, charge | This topic discusses user experiences with Samsung phones on features like battery life and screen quality. |
| 2 | good, product, far, price, phone, quality, condition, really, deal, valu | This topic highlights positive reviews about the product's quality, price, and overall condition. |
| 3 | love, phone, wife, absolutely, son, daughter, note, gift, husband, bought | This topic reflects users' love for Samsung phones as gifts for family members. |
| 4 | great, phone, price, product, condition, fast, deal, quality, value, shipping | This topic expresses positive feedback on the product's condition and shipping speed. |
| 5 | work, perfect, perfectly, fine, expect, new, look, condition, stop, far | The topic appears that users are satisfied with the phone's performance. |

#### **Table XIV:** **NMF Topic Detection for Huawei Brand**

| **Topic for HuaWei** |  |  |
| --- | --- | --- |
| **Topic No** | **Keywords** | **Explanation** |
| 1 | phone, best, use, huawei, battery, like, camera, work, screen, amaze | This topic discusses the overall positive experience with Huawei phones. |
| 2 | good, battery, product, price, camera, life, quality, far, really, phone | This topic highlights positive reviews about the product's battery life, price, and camera quality. |
| 3 | great, phone, price, work, camera, fast, awesome, quality, battery, money | The topic is similar to Topic 2. |
| 4 | love, phone, far, camera, perfect, thing, battery, fast, quality, daughter | This topic expresses users' love for Huawei phones, and also mentions the phone being a perfect gift for family members. |
| 5 | excelente, teléfono, muy, el, producto, la, que, en, buen, lo | This topic discusses positive experiences with Huawei phones by Spanish-speaking users. |

#### **Table XV:** **NMF Topic Detection for Sony Brand**

| **Topic for Sony** |  |  |
| --- | --- | --- |
| **Topic No** | **Keywords** | **Explanation** |
| 1 | phone, sony, use, screen, like, camera, battery, work, really, best | This topic discusses general user satisfaction with Sony phones. |
| 2 | good, phone, price, far, camera, product, quality, performance, overall, like | This topic focuses on the good quality and overall performance of Sony phones, with users appreciating the price and camera. |
| 3 | love, phone, amaze, absolutely, best, new, awesome, big, wife, bought | This topic captures enthusiastic reviews from users who love their Sony phones. |
| 4 | great, phone, camera, price, work, product, far, quality, picture, awesome | The topic is similar to Topic 2. |
| 5 | excellent, product, camera, recommend, seller, item, highly, high, cellphone, sony | This topic discusses highly positive reviews with users mentioning the product being highly recommended and the good experiences with the sellers. |

#### **Table XVI****: MF Topic Detection for Apple Brand**

| **Topic for Apple** |  |  |
| --- | --- | --- |
| **Topic No** | **Keywords** | **Explanation** |
| 1 | phone, battery, come, work, scratch, screen, use, life, iphone, perfect | This topic discusses the physical condition and battery life of Apple phones. |
| 2 | good, far, condition, product, price, quality, deal, buy, value, look | This topic focuses on the positive review of overall product quality and value. |
| 3 | great, work, product, condition, price, phone, far, value, look, recommend | This topic is similar to Topic 2. |
| 4 | love, daughter, perfect, iphone, phone, new, son, worth, condition, problem | This topic captures emotional reviews, with users expressing love for their iPhones and mentioning that they were perfect gifts for family members. |
| 5 | like, new, look, brand, work, product, happy, arrive, iphone, price | This topic reflects user satisfaction with the new and brand-new look of Apple phones. |

#### **Table XVII:** **NMF Topic Detection for Google Brand**

| **Topic for Google** |  |  |
| --- | --- | --- |
| **Topic No** | **Keywords** | **Explanation** |
| 1 | phone, google, pixel, use, battery, screen, best, charge, issue, camera | This topic discusses screen and charging issue on Google Pixel phones |
| 2 | good, price, far, quality, battery, phone, life, value, really, deal | This topic highlights user satisfaction with the overall quality and value. |
| 3 | great, phone, work, camera, price, condition, fast, awesome, amaze, battery | This topic reflects positive reviews about the working condition of camera and battery. |
| 4 | love, phone, pixel, absolutely, camera, really, iphone, thank, gift, feature | This topic captures user affection for their Google Pixel phones. |
| 5 | new, like, work, look, brand, condition, come, phone, scratch, charger | This topic discusses the condition of new Google phones. |

#### **Table XVIII:** **NMF Topic Detection for Asus Brand**

| **Topic for Asus** |  |  |
| --- | --- | --- |
| **Topic No** | **Keywords** | **Explanation** |
| 1 | phone, like, use, asus, work, charge, screen, sim, look, doesnt | This topic discusses Asus phones usage, charging, screen quality, SIM card issues, and the overall look of the phone. |
| 2 | best, phone, price, asus, choice, range, use, battery, life, phon | This topic highlights positive reviews about good battery life and price. |
| 3 | great, phone, feature, money, fast, battery, easy, life, use, apps | This topic reflects satisfaction with the features of Asus phones and apps. |
| 4 | nice, zenfone, mobile, love, design, work, horrible, sound, mediocre, phone | This topic captures mixed reviews about the Asus Zenfone series. |
| 5 | good, quality, price, need, camera, far, day, thing, battery, ive | This topic is similar to Topic 2. |

#### **Table XIX:** **NMF Topic Detection for OnePlus Brand**

| **Topic for OnePlus** |  |  |
| --- | --- | --- |
| **Topic No** | **Keywords** | **Explanation** |
| 1 | phone, oneplus, use, work, screen, camera, battery, come, charge, sim | This topic discusses the overall experience with OnePlus phones. |
| 2 | good, far, phone, product, nice, recommend, beautiful, worth, feature, replacement | This topic highlights user satisfaction with the phone's features, overall quality, and recommendations. |
| 3 | great, phone, price, fast, money, gb, pleased, storage, definitely, recommend | This topic covers positive review on the value for money of OnePlus phones, fast performance, and adequate storage (GB). |
| 4 | love, best, phone, really, fast, ive, far, plus, im, note | This topic reflects strong positive sentiments, with users expressing love for their OnePlus phones, praising their speed and overall performance. |
| 5 | like, new, phone, brand, look, come, condition, arrive, product, happier | This topic focuses on the newness and condition of OnePlus phones upon arrival |

#### **Table XX:** **NMF Topic Detection for Xiaomi Brand**

| **Topic for Xiaomi** |  |  |
| --- | --- | --- |
| **Topic No** | **Keywords** | **Explanation** |
| 1 | phone, use, work, like, best, camera, battery, screen, price, amaze | This topic highlights users' positive experiences with Xiaomi phones. |
| 2 | good, price, product, really, far, quality, battery, performance, camera, life | This topic discusses the overall satisfaction with camera and battery. |
| 3 | excelente, muy, buen, el, precio, producto, teléfono, la, calidad, que | This topic is likely to reflect positive reviews in Spanish. |
| 4 | love, phone, im, new, far, cellphone, awesome, bought, product, perfect | This topic focuses on users expressing their love for their new Xiaomi phones. |
| 5 | great, phone, price, product, work, camera, money, life, fast, quality | This topic is similar to Topic 1. |

## Appendix C Topic-Based Sentiment Scores for Each Brand Using VADER

#### **Table XXI: Topic-Based Sentiment Score for each brand using VADER**

| Brand | Topic | Terms | Average Sentiment | Sentiment Category |
| --- | --- | --- | --- | --- |
| Motorola | 1 | phone, battery, use, like, screen, charge, moto, life, day, new | 0.43 | Neutral |
| Motorola | 2 | good, product, price, far, phone, quality, value, really, camera, condition | 0.55 | Positive |
| Motorola | 3 | great, price, phone, product, value, deal, fast, camera, life, condition | 0.54 | Positive |
| Motorola | 4 | love, phone, son, absolutely, daughter, awesome, bought, perfect, moto, husband | 0.52 | Positive |
| Motorola | 5 | work, verizon, perfectly, fine, perfect, sim, network, didnt, stop, doesn’t | 0.39 | Neutral |
| Nokia | 1 | phone, use, nokia, like, work, window, apps, screen, battery, camera | 0.44 | Neutral |
| Nokia | 2 | good, price, product, phone, quality, work, really, far, buy, look | 0.48 | Neutral |
| Nokia | 3 | great, phone, price, work, product, value, fast, buy, money, awesome | 0.5 | Positive |
| Nokia | 4 | love, phone, window, wife, new, son, bought, perfect, husband, amaze | 0.53 | Positive |
| Nokia | 5 | excellent, product, seller, price, thanks, condition, thank, item, venezuela, delivery | 0.45 | Neutral |
| Samsung | 1 | phone, like, use, new, battery, screen, samsung, nice, come, charge | 0.39 | Neutral |
| Samsung | 2 | good, product, far, price, phone, quality, condition, really, deal, value | 0.48 | Neutral |
| Samsung | 3 | love, phone, wife, absolutely, son, daughter, note, gift, husband, bought | 0.51 | Positive |
| Samsung | 4 | great, phone, price, product, condition, fast, deal, quality, value, shipping | 0.51 | Positive |
| Samsung | 5 | work, perfect, perfectly, fine, expect, new, look, condition, stop, far | 0.4 | Neutral |
| Huawei | 1 | phone, best, use, huawei, battery, like, camera, work, screen, amaze | 0.54 | Positive |
| Huawei | 2 | good, battery, product, price, camera, life, quality, far, really, phone | 0.61 | Positive |
| Huawei | 3 | great, phone, price, work, camera, fast, awesome, quality, battery, money | 0.62 | Positive |
| Huawei | 4 | love, phone, far, camera, perfect, thing, battery, fast, quality, daughter | 0.62 | Positive |
| Huawei | 5 | excelente, telÃ©fono, muy, el, producto, la, que, en, buen, lo | 0.42 | Neutral |
| Sony | 1 | phone, sony, use, screen, like, camera, battery, work, really, best | 0.5 | Positive |
| Sony | 2 | good, phone, price, far, camera, product, quality, performance, overall, like | 0.6 | Positive |
| Sony | 3 | love, phone, amaze, absolutely, best, new, awesome, big, wife, bought | 0.58 | Positive |
| Sony | 4 | great, phone, camera, price, work, product, far, quality, picture, awesome | 0.59 | Positive |
| Sony | 5 | excellent, product, camera, recommend, seller, item, highly, high, cellphone, sony | 0.61 | Positive |
| Apple | 1 | phone, battery, come, work, scratch, screen, use, life, iphone, perfect | 0.32 | Neutral |
| Apple | 2 | good, far, condition, product, price, quality, deal, buy, value, look | 0.42 | Neutral |
| Apple | 3 | great, work, product, condition, price, phone, far, value, look, recommend | 0.46 | Neutral |
| Apple | 4 | love, daughter, perfect, iphone, phone, new, son, worth, condition, problem | 0.47 | Neutral |
| Apple | 5 | like, new, look, brand, work, product, happy, arrive, iphone, price | 0.41 | Neutral |
| Google | 1 | phone, google, pixel, use, battery, screen, best, charge, issue, camera | 0.41 | Neutral |
| Google | 2 | good, price, far, quality, battery, phone, life, value, really, deal | 0.53 | Positive |
| Google | 3 | great, phone, work, camera, price, condition, fast, awesome, amaze, battery | 0.51 | Positive |
| Google | 4 | love, phone, pixel, absolutely, camera, really, iphone, thank, gift, feature | 0.53 | Positive |
| Google | 5 | new, like, work, look, brand, condition, come, phone, scratch, charger | 0.44 | Neutral |
| ASUS | 1 | phone, like, use, asus, work, charge, screen, sim, look, doesnt | 0.52 | Positive |
| ASUS | 2 | best, phone, price, asus, choice, range, use, battery, life, phon | 0.57 | Positive |
| ASUS | 3 | great, phone, feature, money, fast, battery, easy, life, use, apps | 0.65 | Positive |
| ASUS | 4 | nice, zenfone, mobile, love, design, work, horrible, sound, mediocre, phone | 0.57 | Positive |
| ASUS | 5 | good, quality, price, need, camera, far, day, thing, battery, ive | 0.53 | Positive |
| OnePlus | 1 | phone, oneplus, use, work, screen, camera, battery, come, charge, sim | 0.49 | Neutral |
| OnePlus | 2 | good, far, phone, product, nice, recommend, beautiful, worth, feature, replacement | 0.58 | Positive |
| OnePlus | 3 | great, phone, price, fast, money, gb, pleased, storage, definitely, recommend | 0.6 | Positive |
| OnePlus | 4 | love, best, phone, really, fast, ive, far, plus, im, note | 0.62 | Positive |
| OnePlus | 5 | like, new, phone, brand, look, come, condition, arrive, product, happier | 0.54 | Positive |
| Xiaomi | 1 | phone, use, work, like, best, camera, battery, screen, price, amaze | 0.52 | Positive |
| Xiaomi | 2 | good, price, product, really, far, quality, battery, performance, camera, life | 0.61 | Positive |
| Xiaomi | 3 | excelente, muy, buen, el, precio, producto, telÃ©fono, la, calidad, que | 0.32 | Neutral |
| Xiaomi | 4 | love, phone, im, new, far, cellphone, awesome, bought, product, perfect | 0.56 | Positive |
| Xiaomi | 5 | great, phone, price, product, work, camera, money, life, fast, quality | 0.59 | Positive |
